# PKR Binds Enterovirus IRESs, Displaces Host Translation Factors, and Impairs Viral Translation to Enable Innate Antiviral Signaling

**DOI:** 10.1128/mbio.00854-22

**Published:** 2022-06-02

**Authors:** Mikhail I. Dobrikov, Elena Y. Dobrikova, Zachary P. McKay, Jonathan P. Kastan, Michael C. Brown, Matthias Gromeier

**Affiliations:** a Department of Neurosurgery, Duke University Medical School, Durham, North Carolina, USA; Princeton University

**Keywords:** dendritic cell, IRES, MDA5, PKR, TBK1, eIF4G, interferon, poliovirus, translation

## Abstract

For RNA virus families except *Picornaviridae*, viral RNA sensing includes Toll-like receptors and/or RIG-I. Picornavirus RNAs, whose 5′ termini are shielded by a genome-linked protein, are predominately recognized by MDA5. This has important ramifications for adaptive immunity, as MDA5-specific patterns of type-I interferon (IFN) release are optimal for CD4^+^T cell T_H_1 polarization and CD8^+^T cell priming. We are exploiting this principle for cancer immunotherapy with recombinant poliovirus (PV), PVSRIPO, the type 1 (Sabin) PV vaccine containing a rhinovirus type 2 internal ribosomal entry site (IRES). Here we show that PVSRIPO-elicited MDA5 signaling is preceded by early sensing of the IRES by the double-stranded (ds)RNA-activated protein kinase (PKR). PKR binding to IRES stem-loop domains 5–6 led to dimerization and autoactivation, displaced host translation initiation factors, and suppressed viral protein synthesis. Early PKR-mediated antiviral responses tempered incipient viral translation and the activity of cytopathogenic viral proteinases, setting up accentuated MDA5 innate inflammation in response to PVSRIPO infection.

## INTRODUCTION

Plus-sense RNA virus infection releases translation-competent viral RNA (vRNA) into host cytoplasm. This elicits competing processes at vRNAs; engaging translation factors (viral protein synthesis) and attracting vRNA sensors (host innate defense). In wild-type poliovirus (PV) infection, cytotoxic viral protein synthesis prevails in target host cells *in vivo* (1). PV RNAs carry a 5′-genome-linked protein (VPg) that blocks canonical 5′-“cap”-mediated translation (2). Instead, PV initiates protein synthesis via binding of eukaryotic initiation factor (eIF) 4G to the viral internal ribosomal entry site (IRES) (3). The first nonstructural protein released from the nascent PV polyprotein, 2A proteinase (2A^pro^), directs cleavage of eIF4G (4), the pivotal translation initiation scaffold (5). This results in early, lethal host protein synthesis shutoff, while viral IRES-mediated translation proceeds with C-terminal eIF4G cleavage fragments (6). Lethal 2A^pro^-directed cleavages abrogate host innate defenses (7, 8). The PV IRES strategy is evident as lethal infection of lymphoid and intestinal dendritic cells (DCs)/macrophages *in vivo* (1) and primary human DCs/macrophages *in vitro* (9).

PVSRIPO, the type-1 PV (Sabin) vaccine containing a type 2 rhinovirus IRES (10), has shown promise as an immunotherapy in recurrent glioblastoma (11) and recurrent, nonresectable melanoma (12). PVSRIPO is lytic in many glioma cell lines *in vitro* (13); however, overt cytotoxicity was absent in glioma patient *ex vivo* tumor slices or primary human DCs/macrophages (14). Rather, PVSRIPO elicits pervasive, sustained type-I interferon (IFN) signaling in the tumor microenvironment that culminates in enhanced antitumor T cell priming and function (14, 15). Thus, HRV2 IRES exchange may overturn the cytotoxic paradigm of PV, resulting in accentuated host innate antiviral signaling in target cells. PVSRIPO innate inflammation is dominated by tank binding kinase 1 (TBK1)-IFN regulatory factor 3 (IRF3) signaling specific to upstream MDA5 engagement, yielding a unique pattern of sustained type-I IFN (relative to Toll-like receptor [TLR] and STING agonists) (14). Such MDA5-specific patterns of type-I IFN release were shown to elicit optimal stimuli for CD4^+^T cell T_H_1 polarization (16) and CD8^+^T cell responses (17) in the lymphocytic choriomeningitis virus model. Indeed, TBK1-IRF3 signaling was required for antitumor efficacy and potentiation of antitumor T cell function after PVSRIPO therapy in mice (14). The innate response to enteroviruses (EV) in general and the mechanisms of the sustained type-I IFN release pattern orchestrated by MDA5 are not understood.

In this work, we discovered that delayed MDA5 engagement by PVSRIPO is preceded by double-stranded RNA-activated protein kinase (PKR) and adenosine deaminase 1 acting on RNA (ADAR1) sensing of vRNA. PKR bound to IRES stem-loop domains (SLD) 5–6, resulting in dimerization and autophosphorylation of the PKR activation loop. IRES binding of PKR blocked eIF4G recruitment to the IRES *in vitro*, thus potentially competing with viral translation initiation. Blocking PKR activation enhanced PVSRIPO translation only when implemented within 2 h postinfection (hpi), indicating that homodimerization and activation of PKR kinase activity contribute to shaping virus:host relations at the onset. Blocking TBK1 or JAK/STAT signaling elevated viral translation only after ~24 hpi, implicating separate signal transduction pathways in the innate response coordinated by MDA5. Such layered innate sensing may be required for the defense against EVs, whose vRNAs lack 5′ triphosphate (5′ ppp), the defining nonself distinction of most vRNAs.

## RESULTS

### PVSRIPO:host DC relations are shaped by absent eIF4G cleavage and efficient host innate defenses.

**FIG 1:**
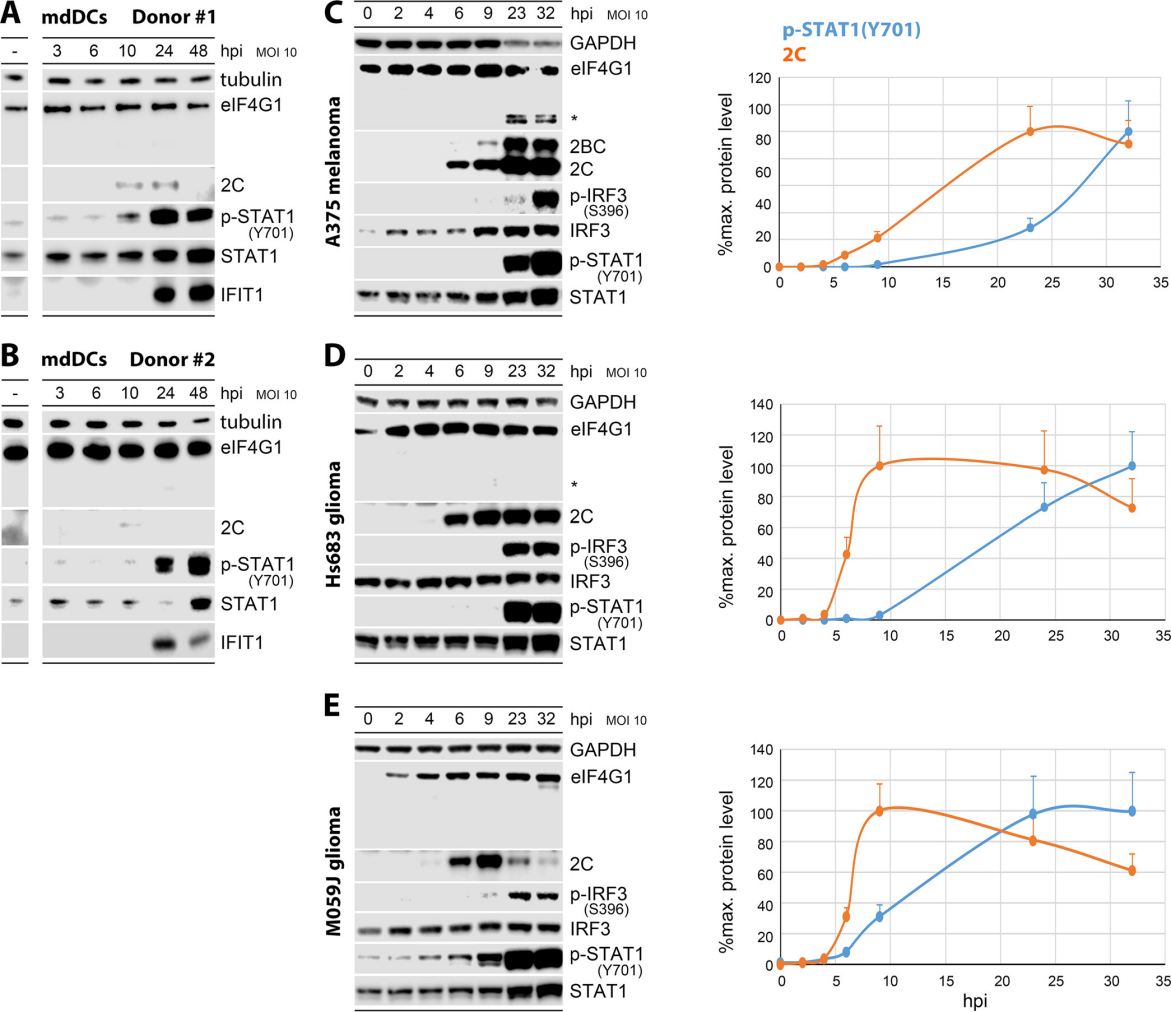
Time course of PVSRIPO infections in primary human monocyte-derived dendritic cells (mdDCs), A375 melanoma, Hs683 glioma, and M059J glioma cells. (A and B) mdDCs from donors 1 (A) and 2 (B) were infected with PVSRIPO and lysates were analyzed by immunoblot for eIF4G1 cleavage, viral (2C) translation, STAT1 activation [p-STAT1(Y701)/STAT1], and ISG induction (IFIT1). (C to E, left) A375 (C), Hs683 (D), and M059J cells (E) were infected with PVSRIPO and lysates were studied by immunoblot for eIF4G1 cleavage (*, eIF4G1 cleavage fragment), viral translation (2C/2BC), IRF3 [p-IRF3(S396)], and STAT1 activation. A minimum of 3 independent series were performed; representative results are shown. (C to E, right) Viral (2C) translation and type I interferon (IFN) signaling [p-STAT1(Y701)] were quantified over a 0–32 hpi period. The values represent the average abundance of signal in 3 independent series (%max. detected, normalized to GAPDH). Standard error of the mean (SEM) is indicated by error bars. MOI, multiplicity of infection; hpi, hours postinfection.

Autopsy studies in humans (18), chimpanzees (19), and macaques (1) show that wild-type PV targets CD11c^+^ DCs/macrophages at a (portal of entry) intestinal site and in lymphoid structures. Histopathology of lymphoid tissues *in vivo* (1) and *in vitro* assays in primary monocyte-derived DCs (mdDCs) revealed cytotoxic wild-type PV infections with rapid host eIF4G cleavage and protein synthesis shut-down (9), preempting innate defenses. PVSRIPO, by virtue of its heterologous HRV2 IRES, has an unusual sublethal phenotype in DCs, with lingering viral propagation (20), absent eIF4G cleavage/host cytopathogenicity (15), and a peculiar sustained type-I/III IFN dominant signature (Fig. 1A and B). PVSRIPO’s specific spatiotemporal IFN pattern is due to MDA5-TBK1-IRF3 driven innate inflammation and is pivotal for antitumor efficacy in mouse tumor models (14). This scenario offers an opportunity to unravel mechanisms of innate anti-EV host defenses orchestrated by MDA5.

To enable proteomic approaches requiring abundant cytoplasmic lysate, we identified A375 melanoma and Hs683 and M059J malignant glioma cell lines that recapitulate the PVSRIPO DC phenotype (Fig. 1C to E). In mdDCs, PVSRIPO (multiplicity of infection [MOI] 10) exhibited low levels of viral translation detected at 10 hpi, which were in decline after 24 hpi, consistent with prior observations (15, 20). There was no indication of eIF4G cleavage in mdDCs after PVSRIPO infection (Fig. 1A and B), as reported previously (15, 20). Viral translation was far more robust in A375, Hs683, and M059J cells; however, as in mdDCs, eIF4G1 cleavage was absent (Hs683, M059J) or inefficient/late (A375) (Fig. 1C to E), without overt cytopathogenicity (Fig. S1). Rather, PVSRIPO elicited signature p-STAT1(Y701) innate signals at similar intervals in all cultures, indicating active host antiviral type-I IFN defenses (Fig. 1A to E). PVSRIPO translation (Fig. 1C and D) and growth (Fig. S1C and D) were productive in A375 and Hs683 cells, despite absent eIF4G1 cleavage and cytopathogenicity, and in the presence of an active innate antiviral type-I IFN response. This confirms prior findings of innate subversion in a melanoma cell line panel not including A375 (21). Of note, p-IRF(S396)/p-STAT1(Y701) signal ramp-up at ~23–32 hpi was ~15–20 h delayed in relation to viral protein synthesis in all models, despite detectable viral progeny at 6 hpi (A375, Hs683 cells; Fig. S1C and D) implying the presence of long double-stranded RNA (dsRNA) replication intermediates in the interval preceding 6 hpi.

10.1128/mbio.00854-22.4 FIG S1 **(related to Fig. 1C and D)** Cytopathogenic effects (CPE) and viral replication in A375 and Hs683 cells infected with PVSRIPO. As a positive control for wild-type enteroviruses (EV) CPE, A375 and Hs683 cells infected with CAV21 are shown as indicated. (A and B) A375 (A) and Hs683 cells (B) were infected with mock, PVSRIPO or CAV21 at multiplicity of infection (MOI) of 10, and the evolution of CPE over time was recorded. (C and D) One-step growth kinetics of PVSRIPO in A375 (C) and Hs683 (D) cells. Average values for each study interval from 3 (C) or 2 (D) independent series were calculated. Download FIG S1, TIF file, 1.0 MB. Copyright © 2022 Dobrikov et al. 2022 Dobrikov et al. https://creativecommons.org/licenses/by/4.0/ This content is distributed under the terms of the Creative Commons Attribution 4.0 International license.

### Host dsRNA sensors are involved in early PVSRIPO detection.

**FIG 2:**
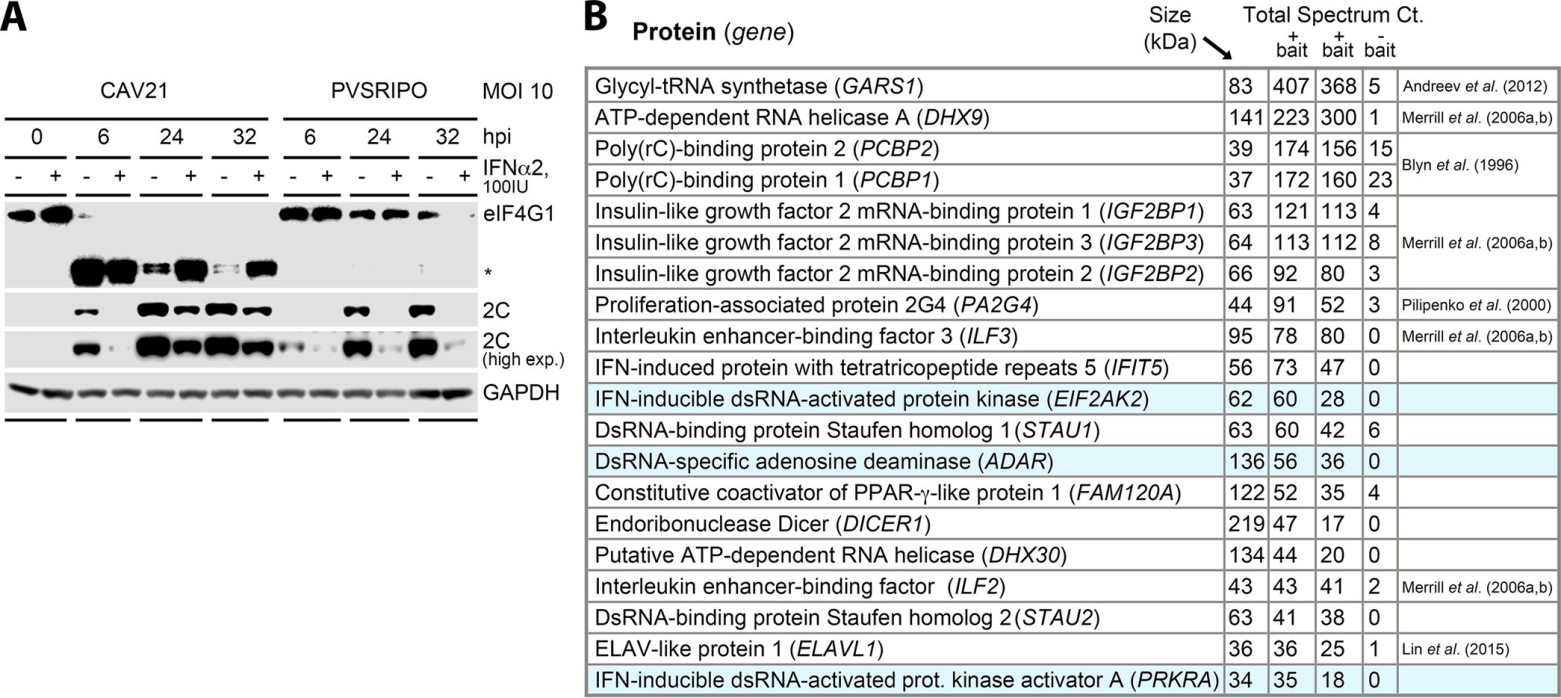
Immediate early innate interference with PVSRIPO involves host double-stranded (ds)RNA sensors interacting with the viral 5′ untranslated region (UTR). (A) A375 cells were pretreated with recombinant human IFNα2 or vehicle (16 h) prior to mock (0 h), CAV21 or PVSRIPO infection. Lysates were collected and analyzed by immunoblot as described for Fig. 1; representative results observed in 3 independent assays. (B) List of proteins of interest identified in our first series of liquid chromatography-mass spectrometry/mass spectrometry (LC-MS/MS) analyses of samples pulled down with PVSRIPO 5′-UTR bait RNA (see Table S2). The list includes all hits that were enriched in both bait^+^ samples with low/absent binding to bait- bead samples (see main text for detail). “Total spectrum count” represents a measure of (bait RNA) binding activity of the protein in question.

PVSRIPO’s inefficient eIF4G1 cleavage and active innate host defense may be due to host interference with early viral translation, e.g., early host sensing of vRNA. To examine this, we tested the effects of challenging A375 cells with IFNα on infection with a wild-type EV with unimpeded eIF4G cleavage and cytotoxicity (Fig. S1A and B) and absent type-I IFN response (coxsackievirus A21 [CAV21] [21]) compared to PVSRIPO (Fig. 2A). IFNα challenge prior to CAV21 infection (16 h) did not prevent eIF4G1 cleavage and delayed viral translation modestly; yet, it abolished PVSRIPO translation (Fig. 2A). Thus, CAV21-mediated immediate early translation and eIF4G cleavage are relatively resistant to type-I IFN responsive factor(s) that suppress PVSRIPO translation. To identify such factors, we used pull down of biotinylated PVSRIPO 5′-untranslated region (UTR) RNA (643 nt; comprising the IRES) as bait. Bait RNA was incubated with cytosolic lysate from A375 cells, followed by pull down with streptavidin beads and liquid chromatography-mass spectrometry/mass spectrometry (LC-MS/MS) analysis (see Materials and Methods).

10.1128/mbio.00854-22.2 TABLE S2 List of proteins of interest identified in our first series of liquid chromatography-mass spectrometry/mass spectrometry (LC-MS/MS) analyses of samples pulled down with PVSRIPO 5′-untranslated region (UTR) bait RNA. Download Table S2, XLSX file, 0.02 MB. Copyright © 2022 Dobrikov et al. 2022 Dobrikov et al. https://creativecommons.org/licenses/by/4.0/ This content is distributed under the terms of the Creative Commons Attribution 4.0 International license.

A comparison of the LC-MS/MS reads for two replicate samples pulled down with bait^+^ versus bait^−^ streptavidin beads yielded 20 proteins that were specifically enriched in both bait^+^ samples without binding to the bait^−^ controls (Table S2). Most of these proteins were previously identified as viral “IRES *trans*-acting factors (ITAFs)”: glycine-tRNA ligase (GARS [22]); poly(rC)-binding protein 2 (PCBP2 [23]); RNA helicase A (DHX9 [24]); insulin-like growth factor 2 mRNA binding protein 1 (IMP-1 [24]); proliferation-associated protein 2G4 (EBP1 [25]); interleukin enhancer-binding factors 3/2 (ILF3 a.k.a. NF90, DRBP76; ILF2 a.k.a. NF45 [24, 26]); and ELAV-like protein 1 (ELAV1 aka. HuR [27]) (Fig. 2B). In addition, the assay isolated several known RNA-binding proteins (Dicer, Staufen1/2, FAM120A) whose interactions with IRESs or involvement in innate antiviral immunity are unclear. A triad of known high-profile innate host dsRNA sensors bound to the 5′-UTR vRNA bait: the dsRNA-activated protein kinase (PKR), the PKR-activating protein (PACT; PRKRA), and the adenosine deaminase acting on RNA 1 (ADAR1) (Fig. 2B). These factors were not previously shown to engage with EV IRESs, potentially due to investigations in systems lacking proficient innate antiviral signaling responses.

**FIG 3:**
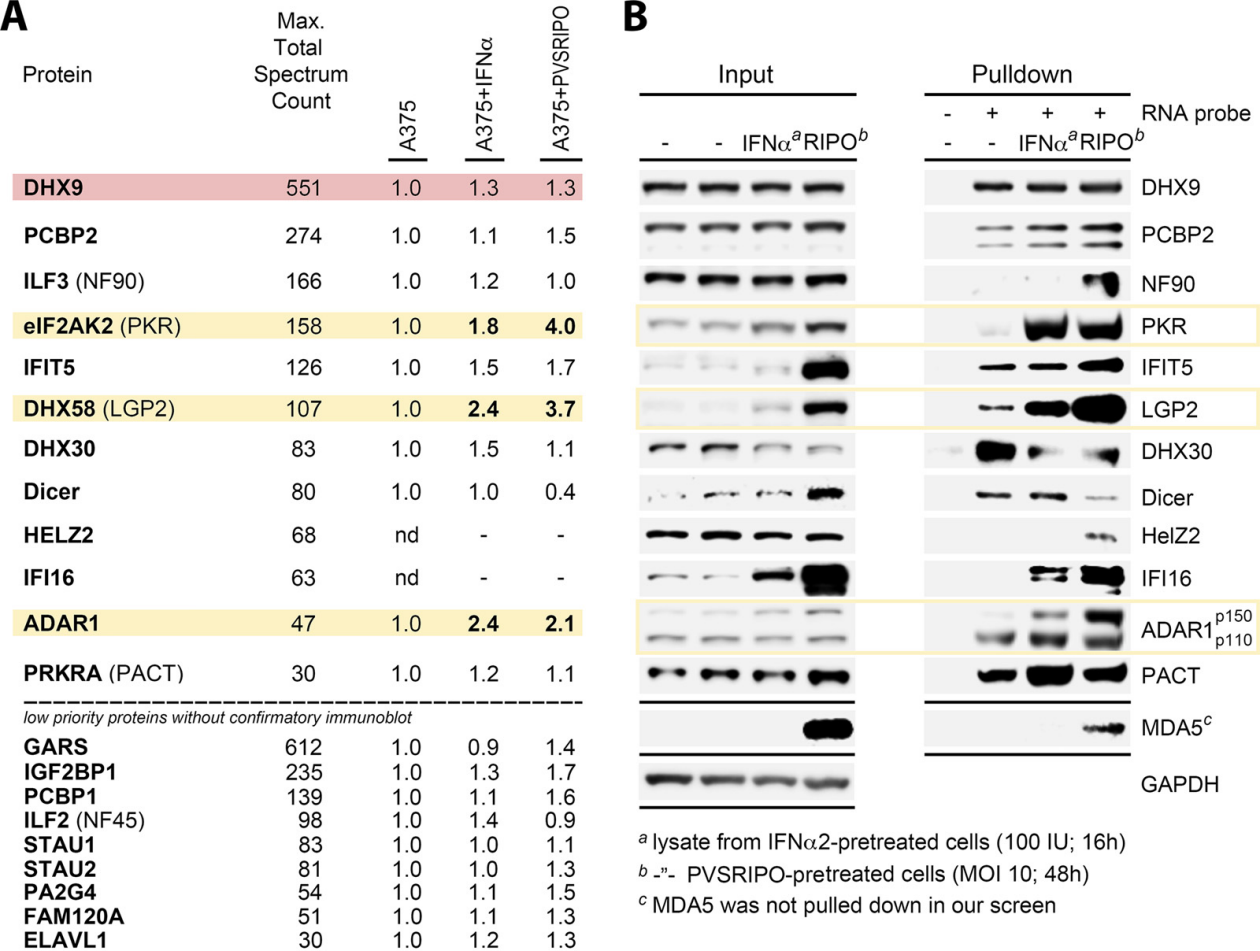
IFN-inducible host innate dsRNA sensors identified to interact with the PVSRIPO 5′ UTR. (A) The top 21 RNA-binding proteins that specifically interacted with the biotinylated RNA bait. Cytosolic fractions of “plain” A375 lysates, or of lysates from A375 cells treated with 100 U/mL recombinant human IFNα2 (16 h) (A375+IFNα), or of lysates from A375 cells treated with PVSRIPO (MOI 10, 48 h) (A375+PVSRIPO) were incubated with biotinylated HRV2 internal ribosomal entry site (IRES) bait (30 min) and pulled down with Streptavidin beads. The pulled down samples were analyzed by LC-MS/MS. Total Spectrum Count represents maximal binding value. Proteins with non IFN-responsive (red) and responsive (yellow) binding, subjected to in-depth analyses, are highlighted. (B) Confirmatory immunoblots of proteins identified by LC-MS/MS (A) in cytosolic fractions of lysates from plain, from IFNα-pretreated, or from PVSRIPO-infected A375; representative results from 3 independent experimental series.

To buttress our findings with more stringent assay conditions, we adapted our approach to probe for host factors that bind vRNA in a type-I IFN-inducible manner. We probed for host protein binding with bait vRNA in plain A375 lysate versus lysates from A375 cells that were pretreated with IFNα or PVSRIPO (Fig. 3A and B). Functionally relevant innate antiviral host protein interactions at the PVSRIPO IRES are expected to be enhanced in IFNα/PVSRIPO primed lysates, since IFNα pretreatment of A375 cells intercepted PVSRIPO translation (Fig. 2A).

The second LC-MS/MS analysis of plain A375 cytosolic lysate baited with biotinylated PVSRIPO 5′-UTR RNA probe essentially corroborated specific binding factors identified in the first series (Table S3; Fig. 3A). Important new information stemmed from analyses of lysate from A375 cells pretreated with IFNα or PVSRIPO (Fig. 3A) and from confirmatory immunoblots of high priority factors identified in our screen (Fig. 3B). First, binding of ITAFs with putative roles in viral translation, i.e., DHX9 (24), PCBP2 (23), or DHX30, was not substantially altered by IFNα/PVSRIPO priming of lysate (Fig. 3A and B). This is expected because these proteins are not induced by type-I IFN (Fig. 3B) or known actors in the innate antiviral defense. The enhanced pull down of NF90 (DRBP76) with bait^+^ beads from PVSRIPO-primed A375 lysate, but not IFNα-primed lysate (Fig. 3B), may be due to cytoplasmic accumulation of DRBP76 in PVSRIPO-infected cells (24). Certain known RNA binding proteins, e.g., HelZ2 or IFI16, only bound to bait in IFNα2 or PVSRIPO-primed lysate without any binding in plain lysate (Fig. 3A and B). Accordingly, they were not identified in the prior proteomic analyses without IFNα/PVSRIPO priming.

10.1128/mbio.00854-22.3 TABLE S3 List of proteins of interest identified in our second series of LC-MS/MS analyses of samples pulled down with PVSRIPO 5′-UTR bait RNA. Download Table S3, XLSX file, 0.04 MB. Copyright © 2022 Dobrikov et al. 2022 Dobrikov et al. https://creativecommons.org/licenses/by/4.0/ This content is distributed under the terms of the Creative Commons Attribution 4.0 International license.

IFNα2/PVSRIPO priming of A375 lysate underscored the potential importance of PKR and ADAR1 binding to bait RNA, first identified in the prior proteomic screen (Fig. 2B). In addition, it revealed LGP2 (DHX58) as a putative interactor with the PVSRIPO 5′ UTR; LGP2 binding was induced with IFNα/PVSRIPO priming (Fig. 3A and B). Indeed, among all LC-MS/MS hits PKR, ADAR1, and LGP2 uniquely shared specific binding to bait vRNA in plain A375 cytosolic lysate that was induced >1.8-fold upon IFNα *and* PVSRIPO-priming (Fig. 3A and B). Therefore, they were pursued as potential actors in innate immune interference of PVSRIPO translation/cytotoxicity.

### Depletion of PKR uniquely enhances early viral translation.

**FIG 4:**
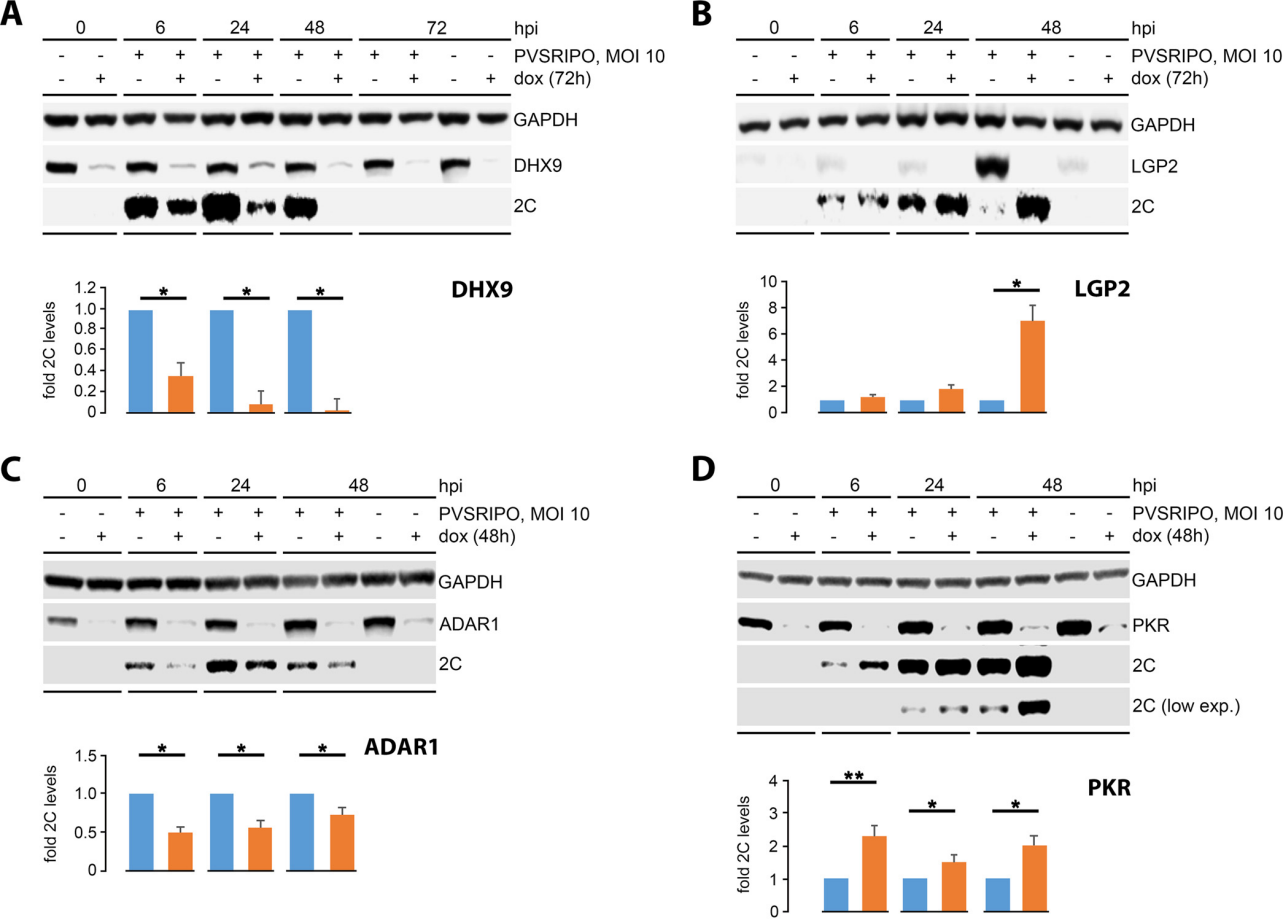
Depletion of double-stranded RNA-activated protein kinase (PKR) enhances early PVSRIPO translation. A375 cells with dox-inducible depletion of DHX9 (A), LGP2 (B), ADAR1 (C), or PKR (D) were infected with PVSRIPO and viral translation was assessed by immunoblot of viral 2C protein. Dox-inducible cell lines were generated using 2 (DHX9, ADAR1, or PKR) or 3 (LGP2) different shRNAs for each depletion target (Table S1). The results shown are from the respective cell lines with more efficient dox-inducible, target-specific depletion. Two series were conducted with each cell line; representative results are shown. Levels of viral 2C in dox-induced (depleted) relative to mock-induced (nondepleted) samples were quantified for each interval in PVSRIPO-infected samples. Graphs represent means ± SE. *, *P* < 0.05, **, *P* < 0.005, respectively.

To evaluate a role in shaping PVSRIPO:host relations for innate dsRNA sensors in our screen, we created doxycycline (dox)-inducible A375 cell lines expressing shRNAs targeting LGP2, PKR or ADAR1 transcripts. We also constructed a cell line with dox-inducible depletion of DHX9, a protein with putative roles in template RNA unwinding/translation (28) and lacking type-I IFN induction (Fig. 4A). Dox-induction (48–72 h) achieved ≥80% depletion of target proteins (Fig. 4A to D). DHX9, in terms of total spectrum count, was a prominent hit in both proteomic analyses (Fig. 2B and 3A), but binding to the PVSRIPO 5’UTR was not enhanced by IFNα/PVSRIPO priming of lysates (Fig. 3A and B). DHX9 depletion caused profound suppression of viral translation (>95% at 48 hpi; Fig. 4A).

10.1128/mbio.00854-22.1 TABLE S1 Oligonucleotide primers used in this study. Download Table S1, TIF file, 1.2 MB. Copyright © 2022 Dobrikov et al. 2022 Dobrikov et al. https://creativecommons.org/licenses/by/4.0/ This content is distributed under the terms of the Creative Commons Attribution 4.0 International license.

Depletion of LGP2, ADAR1, or PKR each revealed modulatory roles in PVSRIPO:host relations, albeit in distinct ways. LGP2 depletion enhanced PVSRIPO translation, but only at intervals with type-I IFN-mediated LGP2 induction (48 hpi; Fig. 4B). Thus, LGP2 requires IFN-mediated priming to generate levels requisite for vRNA engagement, aligning with a role for LGP2 in sustaining MDA5-mediated sensing of picornaviral RNA (29). Due to its late activation profile, LGP2 is unlikely to participate in immediate early innate sensing that intercepts PVSRIPO translation and 2A^pro^-directed host protein cleavages. In contrast to LGP2 depletion, dox-induced ADAR1 depletion suppressed PVSRIPO translation, in accordance with evidence for a role in reigning in MDA5 signaling (30) (Fig. 4C). Only depletion of PKR enhanced early PVSRIPO translation in a manner consistent with a role in modulating early viral translation and 2A^pro^-directed eIF4G1 cleavage (Fig. 4D). Collectively, our data indicate that early PKR:vRNA interactions at the PVSRIPO 5′ UTR may account for the restrained IRES phenotype in mdDCs (Fig. 1A and B) and in A375, Hs683 and M059J cells (Fig. 1C to E), setting up sustained type-I IFN signaling.

### PKR is activated upon binding to HRV2 IRES stem-loop domain 5.

**FIG 5:**
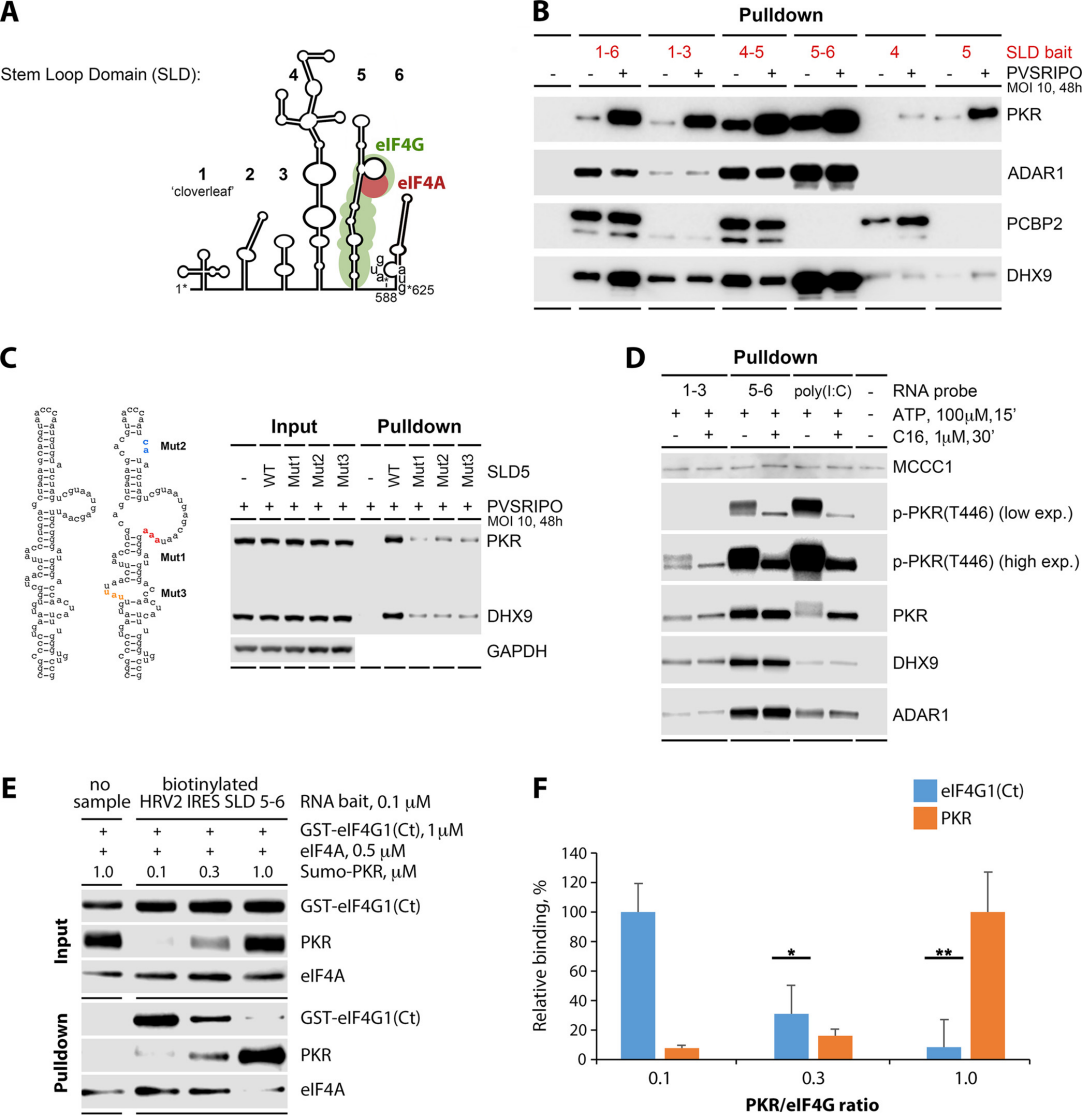
PKR binds to stem-loop domains (SLDs) 5–6 in the HRV2 IRES as a dimer, resulting in autophosphorylation of the kinase activation loop (see Fig. S2 and S3 for extended data). (A) Schematic representation of the HRV2 IRES SLD structure with an approximate footprint for eukaryotic initiation factor (eIF) 4G:eIF4A (3). (B) Binding of PKR, ADAR1, PCBP2, and DHX9 to different SLD fragments as indicated. Cytosolic fractions of A375 cells, “plain” or infected with PVSRIPO (MOI 10, 48 h), were incubated with biotinylated RNA fragments (30 min) prior to pull down with Streptavidin beads. Bound proteins were analyzed by immunoblot (see Fig. S2 for corresponding input blots). (C) PKR and DHX9 binding with biotinylated wild-type (WT) SLD 5 and three distinct SLD 5 variants carrying mutations (Mut 1–3) disrupting base-pairing structure as shown. (D) C16 prevented PKR activation, evident as autophosphorylation of T446 in the kinase activation loop, upon binding to biotinylated SLD 1–3, SLD 5–6, or poly(I·C). Methylcrotonyl-coa carboxylase subunit 1 (MCCC1) nonspecifically associates with streptavidin beads (in the absence of biotinylated RNA bait) and was used as a control to ensure equal loading. The assay was performed twice, and representative results are shown. Figure S3 shows the results of a repeat *in vitro* phosphorylation assay. (E and F) PKR competes with eIF4G for SLD 5–6 binding. The in vitro binding assay was performed as described (see Materials and Methods), keeping constant the concentrations of recombinant GST-eIF4G(Ct), Flag-eIF4A, and biotinylated RNA bait and varying the concentration of His-SUMO-PKR for a PKR/eIF4G ratio ranging from 0.1 to 1. Immunoblots of input and proteins after RNA pull down are shown (E). The assay was performed twice, and representative results are depicted. Relative levels of eIF4G and PRK after pull down were plotted, where maximal binding was set at 100% (F). Graphs represent the averages of 2 independent experiments. *, *P* < 0.05, **, *P* < 0.005.

We performed fine-mapping studies to identify putative IRES stem-loop domains (SLDs) involved in PKR binding (Fig. 5A and B) and mutational analyses of PKR binding to SLD 5 (Fig. 5C) and assessed PKR dimerization/phosphorylation of the PKR activation loop upon binding to the HRV2 IRES *in vitro* (Fig. 5D). First, we constructed bait vRNAs comprising various portions of the PVSRIPO 5′ UTR for pull down (from A375 cell lysates) of key factors identified in our LC-MS/MS screens (Fig. 5A and B). We validated our assay by pull down of PCBP2 with the HRV2 IRES: as reported before (23), PCBP2 specifically bound to IRES SLD 4 (Fig. 5A and B). In alignment with evidence shown in Fig. 4, binding of the ITAFs PCBP2 and DHX9 (proteins with putative roles in translation initiation, but no known involvement in the innate antiviral defense), was not enhanced in A375 lysates primed with PVSRIPO infection (MOI 10, 48 hpi) (Fig. 5B).

10.1128/mbio.00854-22.5 FIG S2 **(related to Fig. 5B)** Immunoblot of A375 lysates used for biotinylated RNA pull down of the (input) factors indicated. Download FIG S2, TIF file, 0.3 MB. Copyright © 2022 Dobrikov et al. 2022 Dobrikov et al. https://creativecommons.org/licenses/by/4.0/ This content is distributed under the terms of the Creative Commons Attribution 4.0 International license.

10.1128/mbio.00854-22.6 FIG S3 **(related to Fig. 5D)** Repeat *in vitro* double-stranded RNA-activated protein kinase (PKR) (T446) phosphorylation assay. Download FIG S3, TIF file, 0.3 MB. Copyright © 2022 Dobrikov et al. 2022 Dobrikov et al. https://creativecommons.org/licenses/by/4.0/ This content is distributed under the terms of the Creative Commons Attribution 4.0 International license.

Substantial PKR binding in plain A375 lysate was detected with SLD 4–5 and SLD 5–6, with the latter exhibiting the highest binding (Fig. 5B). In agreement with LC-MS/MS analyses of IRES binding in IFNα/PVSRIPO-primed lysates (Fig. 3), PKR binding was enhanced when using PVSRIPO-primed lysate (Fig. 5B). Reflecting proteomic data, the pattern of PKR binding to SLD 4–5 and SLD 5–6 was recapitulated by ADAR1, which, however, was not enhanced with PVSRIPO-primed lysate (Fig. 5B). These data indicate that PKR can bind to the PVSRIPO IRES prior to the onset of viral translation, potentially intercepting it.

Based on biochemical RNA mapping, SLD 5 was determined to be the binding site for eIF4G:eIF4A in the PV IRES (3) (Fig. 5A). eIF4G:eIF4A binding to SLD 5 was proposed to induce conformational changes in SLD 6, mediating enhanced eIF4G:eIF4A recruitment to the IRES with a broader interaction domain including SLD 6, indicating that SLD 5 and 6 may be structurally and functionally integrated (3). This is supported by data showing that SLD 5–6 of PVSRIPO jointly control PVSRIPO’s attenuated neurovirulence phenotype (13, 31). Strongest binding of PKR, ADAR1, and DHX9 to SLD 5–6, rather than to SLD 5 alone (Fig. 5B), further emphasizes the potential functional significance of this dual-SLD arrangement.

PKR activation by dsRNA requires a minimum of 30 bp (32, 33), which are present in SLD 5. To examine structure determinants for PKR binding, we probed SLD 5 variants carrying mutations that disrupt the 31-bp stem structure (Fig. 5C). Mut1 and 3 target known interaction sites for eIF4G:eIF4A on the PV IRES (3); Mut2 disrupts the apical portion of SLD 5 (Fig. 5C). Three distinct disruptions of SLD 5-bp architecture each reduced binding of PKR (and DHX9) to SLD 5 (Fig. 5C).

PKR activation requires homodimerization, which triggers autophosphorylation of TT446/451 in the activation loop (34). To examine PKR(T446) autophosphorylation induced by HRV2 IRES SLDs 5–6 binding, we used biotinylated bait RNA pull downs for *in vitro* phosphorylation assays (Fig. 5D). SLD 5–6 were compared to a negative control, to SLD 1–3 (devoid of substantial PKR binding activity in plain A375 lysate; Fig. 5B), and to high-molecular-weight polyinosinic:poly-cytidylic acid [poly(I·C)], an established substrate for PKR binding and autophosphorylation (35) (Fig. 5D). The assay was conducted in the absence or presence of the PKR inhibitor C16. SLD 1–3 did not exhibit substantial PKR binding or autophosphorylation activity (Fig. 5D). PKR binding and p-PKR(T446) levels were similar for SLD 5–6 and poly(I·C); C16 blocked autophosphorylation in both instances (Fig. 5D). Our data show that PKR can bind HRV2 IRES SLD 5–6 as a dimer, in a manner dependent on SLD 5 base-pairing structure, and that, upon binding SLD 5–6, PKR is activated via autophosphorylation.

### PKR competes with eIF4G1 for binding to HRV2 IRES SLD 5–6.

Enhanced PVSRIPO translation upon PKR depletion (Fig. 4D) suggests that PKR homodimers binding to IRES SLD 5–6 interfere with viral translation by impeding eIF4G recruitment. To test this directly, we conducted *in vitro* competition assays, assessing binding of recombinant proteins to biotinylated HRV2 IRES SLD 5–6 by RNA pull down (Fig. 5E). Since recombinant full-length eIF4G1 is intrinsically unstable, we used the recombinant C-terminal eIF4G1 fragment generated by PV 2A^pro^ cleavage [eIF4G(Ct)] eIF4G1(Ct) contains all domains for binding RNA and the translation initiation helicase (eIF4A, eIF4B) (36, 37), is fully competent for ribosome recruitment (38, 39), functions in IRES-mediated translation in wild-type EV-infected cells (6), and is stable after production as a GST-fusion protein in a bacterial expression system (38). The binding assay was carried out in the presence of recombinant Flag-eIF4A, binding partner of eIF4G at the IRES (3) (Fig. 5A). In addition, we used His-SUMO-tagged full-length PKR from a commercial source (see Materials and Methods). eIF4G1(Ct)-binding to HRV2 IRES SLD 5–6 was reduced ~90% in the presence of His-SUMO-PKR at equimolar levels (Fig. 5E and F), indicating that PKR competes with eIF4G1:eIF4A for the same binding site in the HRV2 IRES.

### PKR catalytic activity affects very early viral translation.

**FIG 6:**
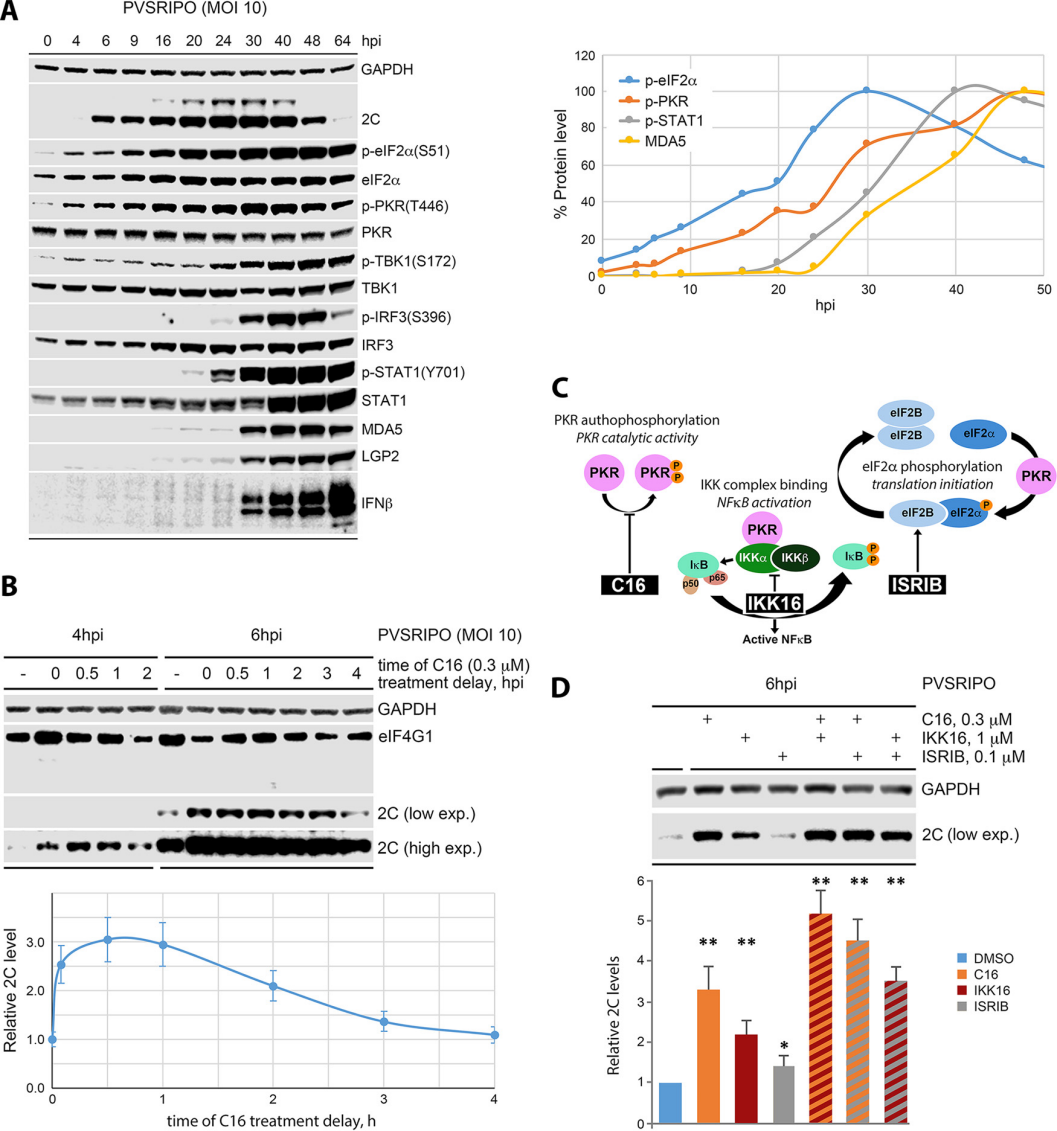
PKR catalytic activity suppresses immediate early PVSRIPO translation (see Fig. S4 and S5 for extended data). (A) Detailed long-range kinetics of the innate antiviral response to PVSRIPO in A375 cells. The dynamics of key innate antiviral signaling cascades were examined by immunoblot (left) and quantitated over time (right). The results of a similar assay in Hs683 cells is shown in Fig. S4. (B) Effect of PKR inhibition with C16 on early viral translation in A375 cells. Cells were infected as shown in Fig. 1 and C16 was added to medium at the time of infection (0-h treatment delay) or at the indicated intervals thereafter. The effect on viral translation was determined by immunoblot of viral 2C at 4 and 6 hpi. The experiment was repeated 3 times; average 2C levels at 6 hpi (relative to mock-treated cells) are graphed at bottom; error bars represent SEM. See Fig. S5 for dose-optimization of small molecule inhibitors used in this study. (C) Schematic representation of inhibitors of PKR downstream signaling: PKR catalytic activity proper (C16), IKKα:β binding/NF-κB activation (IKK16), and the integrated stress response (ISRIB). (D) The effect of C16, IKK16, or ISRIB on early (6 hpi) translation of PVSRIPO in A375 cells. Cells were treated with inhibitors 30 min after PVSRIPO infection and viral translation was assessed by immunoblot of viral 2C. The 2C signal levels were normalized to DMSO-control samples, and relative levels are plotted at the bottom. Graphs represent averages of 3 independent series. *, *P* < 0.05, **, *P* < 0.005.

PKR IRES binding triggers activation of the PKR kinase domain (Fig. 5D). To juxtapose early PKR effects with the host antiviral defense at large, we performed comprehensive analyses of the innate signaling response to PVSRIPO in a 0–64 hpi time course (Fig. 6A). The earliest innate signals detected were p-eIF2α(S51)/p-PKR(T446), evident at 4 hpi (Fig. 6A). In contrast, canonical downstream signals of MDA5/MAVS activation, p-TBK1(S172) and p-IRF3(S396), occurred only at ~24 hpi (Fig. 6A), accounting for late IFNβ synthesis induced by PVSRIPO at 30 hpi. IFN synthesis was sustained, gradually increasing for at least until 64 hpi (Fig. 6A). A similar sustained type-I IFN pattern was observed in PVSRIPO-infected Hs683 glioma cells (Fig. S4).

10.1128/mbio.00854-22.7 FIG S4 **(related to Fig. 6A)** Analysis of the kinetics of the innate antiviral response to PVSRIPO in Hs683 glioma cells. Download FIG S4, TIF file, 0.2 MB. Copyright © 2022 Dobrikov et al. 2022 Dobrikov et al. https://creativecommons.org/licenses/by/4.0/ This content is distributed under the terms of the Creative Commons Attribution 4.0 International license.

**FIG 7:**
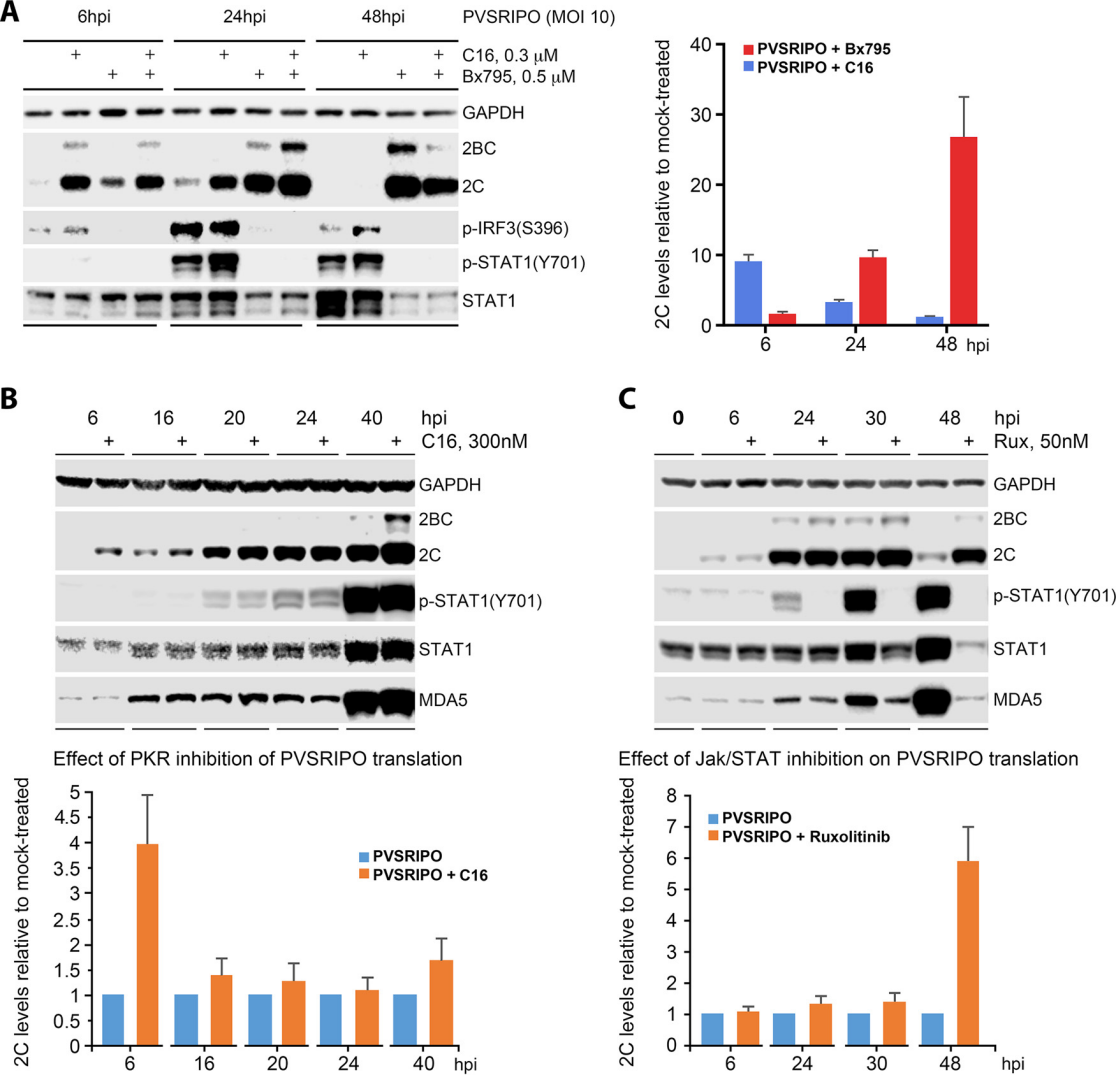
A biphasic innate response to PVSRIPO: early sensing by PKR and a late, polar TBK1-IRF3 inflammatory response orchestrated by MDA5 (see Fig. S6 and S7 for extended data). (A) The effect of TBK1 (Bx795) or PKR (C16) inhibition on early (6 hpi) and late (24, 48 hpi) viral translation in M059J cells, determined by 2C immunoblot. Protein levels at each time point were normalized to corresponding mock-treated samples and plotted in the graph at right. See Fig. S6 for a similar assay in A375 cells. (B and C) Comparing the effect of PKR (C16; B) and JAK/STAT (Ruxolitinib [Rux]; C) inhibition on early (6 hpi) or late (24-48 hpi) viral translation and STAT1 activation in PVSRIPO-infected A375 cells. A375 cells were treated with C16 (B) or Rux (C) at the time of PVSRIPO addition (0 h); viral translation (2C) and STAT1 activation/MDA5 induction were assessed by immunoblot. See Fig. S7 for testing the effect of Rux on PVSRIPO infection of Hs683 cells. All assays were repeated 3 times; representative immunoblots are shown. Relative expression levels of viral 2C were calculated for each time point and are plotted at the bottom.

10.1128/mbio.00854-22.8 FIG S5 **(related to Fig. 6 and 7A and B)** Concentration dependence of PVSRIPO translation (2C) and nonspecific cytotoxicity of pharmacological inhibitors of PKR (C16; A), TBK1 (Bx795; B), and IKKα (IKK16; C) in A375 cells infected with PVSRIPO. A375 cells were incubated PVSRIPO (MOI 10; 30 min), then growth media were substituted with media containing the indicated inhibitors at the concentrations shown. All inhibitors were titrated at concentrations around their IC_50_ (right column) to determine the lowest effective dose for inhibiting the target kinase/signaling pathway with minimal nonspecific cytotoxicity (shown in green font). These concentrations were used for further studies (see main text and related figures). Cell lysates were collected at various intervals post infection and analyzed by immunoblot as indicated. Lysates from mock-infected cultures that were mock-treated or treated with the highest dose of the respective inhibitor were included for each time point to assess effects unrelated to infection. In B, Bx912 was used in parallel with Bx795, because it is a closely related, but not TBK1-specific small molecule inhibitor. In B and C, asterisks indicate cleavage fragments of PARP and eIF4G1, respectively. Download FIG S5, TIF file, 0.5 MB. Copyright © 2022 Dobrikov et al. 2022 Dobrikov et al. https://creativecommons.org/licenses/by/4.0/ This content is distributed under the terms of the Creative Commons Attribution 4.0 International license.

To examine effects of very early PKR signaling on PVSRIPO, we used staggered inhibition with C16 in A375 cells (0.3 μM; see Fig. S5 for dose optimization of C16, IKK16, and Bx795), starting with addition to PVSRIPO infection medium at the 0-h time point (Fig. 6B). C16 enhanced PVSRIPO translation up to a treatment delay of 2 hpi; when added ≥3 hpi, this effect waned (Fig. 6B). PKR signaling effects on very early viral translation are consistent with a PKR activation mode triggered by the IRES (a dsRNA feature of incoming vRNA; Fig. 5D), PKR binding to IRES SLD 5–6 without type-I IFN induction (Fig. 5B), and our finding that PKR depletion enhances viral translation at the onset (Fig. 4D).

Next, we compared inhibitors of documented PKR downstream effectors to test their involvement in the host response to PVSRIPO (Fig. 6C and D). Beyond phosphorylating eIF2α, PKR was implicated in a range of innate inflammatory signals, i.e., STAT1-binding that remains mechanistically unexplored (40), a putative role in p38/JNK MAPK activation (41, 42), association with the IKK complex, through binding IKKβ, involved in NF-κB activation (43–45), or direct interactions of PACT with RIG-I/MDA5 or LGP2 (46, 47) (reviewed in Ref. 48). Given PKR’s involvement in the immediate early host response to PVSRIPO, a role for the IKK complex in this phenomenon was the most plausible. We used inhibitors of PKR catalytic activity (C16), IKKα:β (IKK16), and the integrated stress response (ISRIB) to interrogate early viral translation at 6 hpi (Fig. 6C). Reversing the effects of eIF2α(S51) phosphorylation and restoring translation (ISRIB) barely affected viral translation, consistent with earlier reports (49). Inhibiting NF-κB signaling with IKK16 induced viral translation at 6 hpi ~2-fold (Fig. 6D). The effect was inferior to that of C16 (~3.5-fold) and combining IKK16 with C16 showed additive induction of viral translation (Fig. 6D). This suggests that the effect of IKK16 on viral translation occurs independent of PKR activation.

### Immediate early host sensing of vRNA by PKR sets up a sustained type-I/III IFN response orchestrated by MDA5.

The PVSRIPO host response involves at least two innate modules: PKR and MDA5/TBK1 (Fig. 6A). We used C16 and a TBK1 inhibitor (Bx795) to test possible mechanistic relations of these distinct innate networks in infected M059J glioma cells. Consistent with prior reports of polar TBK1-IRF3 signaling elicited by PVSRIPO (14), Bx795 abolished the canonical type-I IFN host response [p-STAT1(Y701) at 24–48 hpi] to PVSRIPO, stimulating viral translation ~30-fold at 48 hpi (Fig. 7A). Adding Bx795 to C16 did not alter the enhancement of early (6 hpi) viral translation caused by inhibiting PKR (Fig. 7A). This indicates that early interference with viral translation upon innate sensing of PVSRIPO RNA by PKR occurs independently of MDA5/TBK1 signaling. To corroborate our findings, we next compared the effect of inhibiting PKR (C16; Fig. 7B) with inhibiting JAK/STAT signaling (Ruxolitinib [Rux]; Fig. 7C) on PVSRIPO dynamics in A375 cells. In agreement with other data obtained in this study, substantial effects on viral translation upon PKR inhibition occurred at 6 hpi only (Fig. 7B). Rux completely blocked STAT1 signaling but had no effect on viral translation before 48 hpi, when 2C expression was induced ~6-fold relative to the DMSO treated control (Fig. 7C). A similar response to Rux was observed in PVSRIPO-infected Hs683 cells (Fig. S7). Our findings suggest that PVSRIPO host sensing involves separate innate networks that coalesce for an orchestrated type-I IFN response.

10.1128/mbio.00854-22.10 FIG S7 **(related to Fig. 7C)** Immunoblot of viral (2C) translation and markers of the host innate antiviral type-I IFN response in Hs683 glioma cells treated with DMSO (mock) or ruxilitinib (Rux) as indicated. Download FIG S7, TIF file, 0.3 MB. Copyright © 2022 Dobrikov et al. 2022 Dobrikov et al. https://creativecommons.org/licenses/by/4.0/ This content is distributed under the terms of the Creative Commons Attribution 4.0 International license.

10.1128/mbio.00854-22.9 FIG S6 **(related to Fig. 7A)** Immunoblot of viral (2C) translation after inhibition of A375 cells with DMSO (mock), C16, or Bx795. See Fig. 7A and related text/legend for details. Download FIG S6, TIF file, 0.3 MB. Copyright © 2022 Dobrikov et al. 2022 Dobrikov et al. https://creativecommons.org/licenses/by/4.0/ This content is distributed under the terms of the Creative Commons Attribution 4.0 International license.

## DISCUSSION

Innate antiviral responses yield differentiated spatiotemporal patterns of proinflammatory cytokine release coordinated by a vast innate defense system. Apart from a central role of MDA5 (50) innate immunity to EVs is poorly understood. Yet, their peculiar genomes (5′ VPg, the IRES), a drastic tactic to foil host defenses (lethal 2A^pro^-directed host protein cleavage [4, 7]), and eminent clinical importance call for elucidating EV innate immunity. In this work, we deciphered mechanisms of accentuated innate signaling elicited by the highly attenuated PV recombinant PVSRIPO. In contrast to wild-type PVs, which cause cytotoxic eIF4G cleavage in DCs/macrophages (9), PVSRIPO infection is nonlethal and preserves eIF4G (14, 15, 20). This host phenotype elicits a peculiar innate antiviral signature in myeloid antigen-presenting cells, dominated by polar TBK1-IRF3 signaling and sustained type-I/III IFN release (14, 15).

PVSRIPO’s attenuated 2A^pro^-directed cleavages, enabling innate defenses to set in, implicate host innate sensors interfering with immediate early viral translation. Because EV RNAs lack 5′ ppp, the prototypical foreign pattern of vRNAs, early sensing may rely on detecting dsRNA structures in incoming EV genomes. The onset of PVSRIPO induced MDA5-TBK1-IRF3 signaling did not occur before ~20 hpi, after reaching peak viral replication, excluding MDA5 as a factor in early sensing of vRNA. Proper activation of MDA5 may require its induction, LGP2 assistance, and the accumulation of ds vRNA intermediates, accounting for this delay.

Biotinylated RNA pull down of cytoplasmic lysates followed by LC-MS/MS analysis revealed innate dsRNA sensors PKR, ADAR1, and LGP2 binding to the viral IRES, which was substantially enhanced with lysate from IFNα/PVSRIPO-pretreated cells. Depletion studies revealed that only PKR negatively regulated early viral translation. RNA pull down for mapping of IRES domains demonstrated that PKR binds to IRES SLD 5–6, the same structure responsible for translation initiation via recruitment of the eIF4G:eIF4A translation initiation helicase (3). Our studies revealed that beyond eIF4G:eIF4A and PKR, IRES SLD 5–6 attract other innate dsRNA sensors (e.g., ADAR1) and translation factors (eg. DHX9) directly.

*In vitro* phosphorylation studies with SLD 5–6 pull down demonstrated that PKR binds the IRES as a dimer, inducing autophosphorylation of its activation loop. PKR displaced eIF4G:eIF4A recruitment *in vitro*, indicating that PKR homodimer binding to SLD 5–6 may account for early interference with PVSRIPO translation. Moreover, inhibiting PKR signaling with C16 enhanced viral translation, indicating that PKR activation plays a role in downregulating viral translation. This effect only occurred when C16 was added 0–2 hpi, consistent with our observations of PKR-IRES binding acting on immediate early viral translation. In contrast, inhibitors of TBK1 or JAK/STAT enhanced viral translation not before 24 or 48 hpi, respectively.

Our studies suggest that PKR interactions with the IRES inhibit viral growth in initial stages of the infection, operating through signaling pathways distinct from the canonical response orchestrated by MDA5. Such interference with early viral translation may prevent 2A^pro^-directed host protein cleavages and host cell pathogenicity and enable a full-fledged innate antiviral type-I IFN response. A “staggered” innate defense, involving layered dsRNA sensing by distinct innate modules acting on discrete stages of the infection, may be key to tailor innate defenses to the specific viral challenge at hand.

The shared PKR and eIF4G:eIF4A footprint in IRES SLD 5, enabling viral cap-independent translation initiation, is highly conserved in EVs (3). Our data indicate that distinct cytotoxicity of PVSRIPO versus wild-type PV, e.g., in human DCs, depends on the balance of host dsRNA-binding assemblies forming at IRES SLD 5–6 early after infection. Both eIF4G:eIF4A (3) and PKR bind more efficiently to SLD 5–6 than SLD 5 alone, indicating a role of higher order conformational arrangements in the 3′ portion of the IRES. These may differ in rhinovirus versus PV IRESs, as the apical regions of their SLDs 5 and 6 diverge (10, 31), possibly affecting the capacity for recruiting eIF4G:eIF4A *in vivo*. This is difficult to test empirically since eIF4G IRES binding cannot be assessed in this setting. Moreover, host dsRNA sensors and translation machinery are substrates of host cell signaling cascades, accounting for cell type-specific cytopathogenicity. For example, PKC-Raf-ERK1/2 mitogenic signaling to eIF4G favors viral IRES-mediated translation by stimulating 40S subunit recruitment (36, 51, 52). Indeed, lacking PVSRIPO cytopathogenicity in HEK293 cells was restored upon transduction with oncogenic Ras (53).

Despite relying on eIF2 for translation via the IRES (3), and in contrast to most viral pathogens, EVs do not possess known countermeasures to PKR activation. They exploit a privileged translation compartment at the ER, shielded from PKR-mediated eIF2α(S51) phosphorylation (49). This relationship is evident in the present study, where productive viral translation and replication take place, despite PKR activation/eIF2α(S51) phosphorylation, and in the absence of eIF4G cleavage. Indeed, abundant viral translation and replication in the presence of a full-blown innate antiviral response confirms the capacity of EVs for evading innate host immunity by virtue of their simplistic, highly efficient replication strategy (54).

The innate nexus identified in our study to interact with the HRV2 IRES also was shown to respond to paired Alu retroelement (Alu:Alu) hybrids, present in 3′ UTRs of pol II transcripts in primates (55, 56). Similar to EVs, host sensing of such endogenous cytoplasmic dsRNA centers on MDA5 (55). We discovered opposing functions for PKR and ADAR1 in the innate response to PVSRIPO, consistent with RNA-editing independent roles for ADAR1 in tempering PKR activation by Alu retroelements (56) and in response to RNA virus infection (reviewed in Ref. 57). Thus, the innate system sensing endogenous cytoplasmic dsRNA in primates may have its origin in the evolved defense against picornavirus infection.

## MATERIALS AND METHODS

### Cell lines and doxycycline-inducible cell lines.

HeLa R19, A375, and Hs683 cells were maintained in high-glucose DMEM supplemented with 0.1 mM nonessential amino acids (Invitrogen) and 10% fetal bovine serum (FBS; Sigma). M059J cells were grown in DMEM:F12 (Invitrogen) supplemented with 0.05 mM nonessential amino acids and 10% FBS. To generate stable cell lines with dox-inducible knockdown of genes of interest, A375 cells were first transfected with BstZ17I-digested pcDNA6/TR (Invitrogen), the tetracycline repressor expression vector, and grown under blasticidin (5 μg/mL; Invitrogen) selection. Blasticidin-resistant clones were screened for dox-inducible *RLuc* expression after transient transfection with pcDNA5/TO/FRT/*Luc* (38), and the clone with the lowest *RLuc* basal level:highest induction ratio was selected and expanded as a host cell line. Stable cell lines with dox-inducible knockdown of *ADAR*, *DHX9*, *DHX58* (LGP2), or *EIF2AK2* (PKR) were generated with shRNA-expressing vectors (see below); G418-resistant (600 μg/mL; Invitrogen) clones were screened by immunoblot of lysates from control/dox-induced cells (1 μg/mL; 48–72 h) for the highest level of target protein depletion. MdDCs were generated from leukopak-derived PBMCs (Stemcell Tech) by allowing monocytes to adhere (1 h) at 37°C in AIM-V media (Thermo Fisher), followed by 6 days of culture in 50 μg/mL GMCSF (R&D systems) and 20 ng/mL IL-4 (Stemcell Tech).

### Viruses.

PVSRIPO used in this work is from a good-laboratory-practice lot prepared for research purposes only. Briefly, infectious cDNA plasmid (10) was linearized with MluI, used as template for T7 *in vitro* transcription (Megascript, Thermo Fisher) to generate full-length vRNA. *In vitro* transcript RNA was transfected into HeLa R19 cells using DMRIE-C in Opti-MEM (Thermo Fisher) and virus recovered from the transfection procedure was titered by plaque assay (10). To prepare virus stocks, four 10-cm dishes of HeLa cells at ~90% confluence were infected at an MOI 10 and incubated 16–24 h until complete CPE. Following three freeze thaw cycles, the culture medium was collected, centrifuged at 14,000 × *g* and the resulting supernatant filtered through 0.1-μM syringe filters (Pall Corp.). Virus suspension was concentrated by centrifugation through a 100-kDa cutoff spin column (Millipore). Viral stocks were titered by plaque assay (10). CAV21 was propagated/purified using similar procedures (21).

### Cloning and *in vitro* transcription.

ADAR1-, DHX9-, LGP2-, and PKR-specific shRNAs were designed according to validated Mission shRNA clones (Sigma-Aldrich) (Table S1) and cloned into pcDNA3.1/TO replacing miR-4G (58). At least two different shRNAs were tested for each gene. Individual/dual HRV IRES SLDs were PCR amplified using primers (Table S1) and cloned into NheI-XbaI sites of the pTNT vector (Promega). Mutations in SLD 5 were introduced with QuikChange Lightning kit (Agilent) using primers specified in Table S1. *In vitro*-transcribed RNA fragments (MEGAscript T7 Kit; Thermo Fisher) were trace labeled with biotin-16-UTP (Roche).

### Inhibitors, antibodies, and immunoblot.

PKR inhibitor C16 (Tocris), IKKα:β inhibitor IKK16, TBK1 inhibitor Bx795 and JAK/STAT inhibitor Ruxolitinib (all Selleck Chemicals), and ISR inhibitor ISRIB (Sigma) were dissolved in DMSO and used at the indicated concentrations. Biotinylated HMW poly(I·C) was purchased from InvivoGen and recombinant IFNα2 was from PBL Assay Science. Primary antibodies used in this study were against eIF4G1, eIF4A, GAPDH, IRF3, p-IRF3(S396), STAT1, p-STAT1(Y701), IFNβ, IFIT1, PCBP2, PKR, ADAR, Dicer, IFI16, PACT, MDA5, TBK1, p-TBK1(S172), eIF2a, p-eIF2α(S51) (all Cell Signaling Technology), DHX9, LGP2, IFIT5 (all Proteintech), HelZ2, MCCC1 (ThermoFisher), DHX30 (Novus), NF90 (BD Biosciences), α-tubulin (Sigma-Aldrich), and p-PKR(T446) (LSBio). Anti-2C antibody was a gift from E. Wimmer (Stony Brook Univ., NY). Immunoblots were performed as described previously (59, 60).

### Cell fractionation, streptavidin pull down, and LC-MS/MS analyses.

Cell lysates for immunoblot in time course assays were prepared essentially as before (59, 60). To inhibit tyrosine phosphatases, one additional wash with PBS containing 100 μM sodium orthovanadate (Sigma) was performed before cell lysis. To isolate the cytosolic fraction, melanoma A375 cells were grown in 150-mm dishes to 80–90% confluence, scraped into detergent-free PBS and centrifuged at 500 × *g* for 5 min at 4°C. Cell pellets were resuspended in 0.5 mL per dish solubilization buffer containing 20 mg/mL n-dodecyl-b-d-maltoside (Calbiochem) and incubated at 4°C for 30 min. After centrifugation at 10,000 × *g* for 10 min, the supernatant containing cytosolic proteins was collected and incubated with biotinylated RNA for 15 min at 4°C (5 μg of PVSRIPO 5′-UTR RNA or equimolar amounts of other dsRNAs were used per 1 mL lysate). RNA pull down was performed for 1.5 h at 4°C with 150 μL streptavidin-Sepharose beads (GE Healthcare) preblocked with 1% BSA in NT2 buffer for 1.5 h. Beads with bound RNA-protein complexes were washed four times with 0.5 mL NT2 buffer containing RNaseOUT/Halt protease inhibitor (both ThermoFisher) and subjected to immunoblotting or quantitative LC-MS/MS analysis performed at Duke University Proteomics facility (see below).

### *In vitro* phosphorylation assay.

*In vitro* phosphorylation of PKR bound to various dsRNAs was performed on beads after streptavidin pull down. Bound complexes were treated with protein phosphatase λ (NEB; 30 min, 30°C) in PMP buffer, and reactions were stopped by addition of 2 mM sodium orthovanadate (10 min, 30°C). Then, the beads were washed twice with 0.5 mL kinase buffer (20 mM HEPES pH 7.4, 4 mM MgCl_2_, 50 mM KCl, 2 mM DTT, and RNase/protease inhibitor) and split into three reactions: 100 μM ATP (15 min, 30°C) with or without C16 pretreatment (1 μM; 15 min) and without ATP (negative control). Finally, samples were separated by SDS-PAGE on NuPAGE 8% Bis-Tris gels (Life Technologies) and probed with the indicated antibodies.

### *In vitro* competition assay.

Recombinant GST-tagged C-terminal fragment (Ct) of eIF4G1 was expressed and purified as described earlier (38). Recombinant full-length His-SUMO-PKR and Flag-eIF4A1 were purchased from LifeSpan BioSciences or EUPROTEIN Inc., respectively. Biotinylated RNA fragment of HRV2 IRES (SLD 5–6) was used as a binding bait. Each competition reaction contained 1 μM GST-eIF4G(Ct), 0.5 μM Flag-eIF4A1, 0.1 μM bait RNA, and increasing concentrations of His-SUMO-PKR (0.1–0.3–1.0 μM). Protein mix without RNA was used as a negative control. First, biotinylated RNA was incubated with GST-eIF4G(Ct):Flag-eIF4A1 (5 min) at 20°C to preassemble complexes. Next, His-SUMO-PKR was added for another incubation period (5 min), followed by RNA pull down on streptavidin magnetic beads (New England BioLabs) and immunoblot analysis.

### Statistical analyses.

Quantification of immunoblot signal was performed using the Li-COR Odyssey FC imaging system and Image Studio software. All experiments were repeated at least three times. Normalization methods for quantified immunoblot signals are described in the figure legends; average values and standard error of the means are represented. Paired *t* test was used for comparison of two different conditions, and significance was defined as a *P* value of <0.05 as described in the figure legends.

### Proteomics.

LC-MS/MS analyses of proteins bound to dsRNA were performed at the Duke Proteomics facility. Briefly, samples were spiked with 120 fmol bovine casein and supplemented with 15 μL 20% SDS, reduced with 10 mM dithiothreitol (30 min) at 80°C and alkylated with 20 mM iodoacetamide (30 min) at 20°C. Samples were supplemented with a final concentration of 1.2% phosphoric acid and 555 μL of S-Trap (Protifi) binding buffer (90% MeOH/100 mM TEAB). Proteins were trapped on the S-Trap, digested using 20 ng/μL sequencing grade trypsin (Promega) for 1 h at 47°C, and eluted using 50 mM TEAB, followed by 0.2% FA, and lastly using 50% ACN/0.2% FA. All samples were then lyophilized to dryness and resuspended in 12 μL 1%TFA/2% acetonitrile containing 12.5 fmol/μL yeast alcohol dehydrogenase (ADH_YEAST).

Quantitative LC-MS/MS was performed using a nanoAcquity UPLC system (Waters Corp) coupled to a Thermo Orbitrap Fusion Lumos high-resolution accurate mass tandem mass spectrometer (Thermo) via a nano-electrospray ionization source. Briefly, the sample was first trapped on a Symmetry C18 20 mm × 180 μm trapping column (5 μL/min at 99.9/0.1 vol/vol water/acetonitrile), after which the analytical separation was performed using a 1.8 μm Acquity HSS T3 C18 75 μm × 250 mm column (Waters Corp.) with a 90-min linear gradient of 5% to 30% acetonitrile with 0.1% formic acid at a flow rate of 400 nL/min with a column temperature of 55°C. Data collection on the Fusion Lumos mass spectrometer was performed in a data-dependent acquisition mode of acquisition with a *r* = 120,000 (@ *m/z* 200) full MS scan from *m/z* 375 to 1,500 with a target AGC value of 2e5 ions. MS/MS scans were acquired at Rapid scan rate (Ion Trap) with an AGC target of 5e3 ions and a max injection time of 100 ms. The total cycle time for MS and MS/MS scans was 2 sec. A 20-sec dynamic exclusion was employed to increase depth of coverage. The total analysis cycle time for each sample injection was approximately 2 h.

## References

[1] Shen L, Chen CY, Huang D, Wang R, Zhang M, Qian L, Zhu Y, Zhang AZ, Yang E, Qaqish A, Chumakov K, Kouiavskaia D, Vignuzzi M, Nathanson N, Macadam AJ, Andino R, Kew O, Xu J, Chen ZW. 2017. Pathogenic events in a nonhuman primate model of oral poliovirus infection leading to paralytic poliomyelitis. J Virol 91:e02310-16. doi:10.1128/JVI.02310-16. 28356537 PMC5487571

[2] Lee YF, Nomoto A, Detjen BM, Wimmer E. 1977. A protein covalently linked to poliovirus genome RNA. Proc Natl Acad Sci USA 74:59–63. doi:10.1073/pnas.74.1.59. 189316 PMC393196

[3] de Breyne S, Yu Y, Unbehaun A, Pestova TV, Hellen CU. 2009. Direct functional interaction of initiation factor eIF4G with type 1 internal ribosomal entry sites. Proc Natl Acad Sci USA 106:9197–9202. doi:10.1073/pnas.0900153106. 19470487 PMC2695064

[4] Etchison D, Milburn SC, Edery I, Sonenberg N, Hershey JW. 1982. Inhibition of HeLa cell protein synthesis following poliovirus infection correlates with the proteolysis of a 220,000-dalton polypeptide associated with eucaryotic initiation factor 3 and a cap binding protein complex. J Biol Chem 257:14806–14810. doi:10.1016/S0021-9258(18)33352-0. 6294080

[5] Hentze MW. 1997. eIF4G: a multipurpose ribosome adapter? Science 275:500–501. doi:10.1126/science.275.5299.500. 9019810

[6] Dobrikova EY, Grisham RN, Kaiser C, Lin J, Gromeier M. 2006. Competitive translation efficiency at the picornavirus type 1 internal ribosome entry site facilitated by viral cis and trans factors. J Virol 80:3310–3321. doi:10.1128/JVI.80.7.3310-3321.2006. 16537598 PMC1440366

[7] Kastan JP, Tremblay MW, Brown MC, Trimarco JD, Dobrikova EY, Dobrikov MI, Gromeier M. 2021. Enterovirus 2A(pro) cleavage of the YTHDF m(6)A readers implicates YTHDF3 as a mediator of type i interferon-driven JAK/STAT signaling. mBio 12:e00116-21. doi:10.1128/mBio.00116-21. 33849973 PMC8092205

[8] Morrison JM, Racaniello VR. 2009. Proteinase 2Apro is essential for enterovirus replication in type I interferon-treated cells. J Virol 83:4412–4422. doi:10.1128/JVI.02177-08. 19211759 PMC2668472

[9] Wahid R, Cannon MJ, Chow M. 2005. Dendritic cells and macrophages are productively infected by poliovirus. J Virol 79:401–409. doi:10.1128/JVI.79.1.401-409.2005. 15596833 PMC538697

[10] Gromeier M, Alexander L, Wimmer E. 1996. Internal ribosomal entry site substitution eliminates neurovirulence in intergeneric poliovirus recombinants. Proc Natl Acad Sci USA 93:2370–2375. doi:10.1073/pnas.93.6.2370. 8637880 PMC39803

[11] Desjardins A, Gromeier M, Herndon JE, Beaubier N, Bolognesi DP, Friedman AH, Friedman HS, McSherry F, Muscat AM, Nair S, Peters KB, Randazzo D, Sampson JH, Vlahovic G, Harrison WT, McLendon RE, Ashley D, Bigner DD. 2018. Recurrent glioblastoma treated with recombinant poliovirus. N Engl J Med 379:150–161. doi:10.1056/NEJMoa1716435. 29943666 PMC6065102

[12] Beasley GM, Nair SK, Farrow NE, Landa K, Selim MA, Wiggs CA, Jung SH, Bigner DD, True Kelly A, Gromeier M, Salama AK. 2021. Phase I trial of intratumoral PVSRIPO in patients with unresectable, treatment-refractory melanoma. J Immunother Cancer 9:e002203. doi:10.1136/jitc-2020-002203. 33875611 PMC8057552

[13] Gromeier M, Lachmann S, Rosenfeld MR, Gutin PH, Wimmer E. 2000. Intergeneric poliovirus recombinants for the treatment of malignant glioma. Proc Natl Acad Sci USA 97:6803–6808. doi:10.1073/pnas.97.12.6803. 10841575 PMC18745

[14] Brown MC, Mosaheb MM, Mohme M, McKay ZP, Holl EK, Kastan JP, Yang Y, Beasley GM, Hwang ES, Ashley DM, Bigner DD, Nair SK, Gromeier M. 2021. Viral infection of cells within the tumor microenvironment mediates antitumor immunotherapy via selective TBK1-IRF3 signaling. Nat Commun 12:1858. doi:10.1038/s41467-021-22088-1. 33767151 PMC7994570

[15] Brown MC, Holl EK, Boczkowski D, Dobrikova E, Mosaheb M, Chandramohan V, Bigner DD, Gromeier M, Nair SK. 2017. Cancer immunotherapy with recombinant poliovirus induces IFN-dominant activation of dendritic cells and tumor antigen-specific CTLs. Sci Transl Med 9:eaan4220. doi:10.1126/scitranslmed.aan4220. 28931654 PMC6034685

[16] De Giovanni M, Cutillo V, Giladi A, Sala E, Maganuco CG, Medaglia C, Di Lucia P, Bono E, Cristofani C, Consolo E, Giustini L, Fiore A, Eickhoff S, Kastenmuller W, Amit I, Kuka M, Iannacone M. 2020. Spatiotemporal regulation of type I interferon expression determines the antiviral polarization of CD4(+) T cells. Nat Immunol 21:321–330. doi:10.1038/s41590-020-0596-6. 32066949 PMC7043938

[17] Wang Y, Swiecki M, Cella M, Alber G, Schreiber RD, Gilfillan S, Colonna M. 2012. Timing and magnitude of type I interferon responses by distinct sensors impact CD8 T cell exhaustion and chronic viral infection. Cell Host Microbe 11:631–642. doi:10.1016/j.chom.2012.05.003. 22704623 PMC3572910

[18] Burrows MT. 1931. Is poliomyelitis a disease of the lymphatic system? Arch Intern Med 48:33–50. doi:10.1001/archinte.1931.00150010038002.

[19] Bodian D. 1955. Emerging concept of poliomyelitis infection. Science 122:105–108. doi:10.1126/science.122.3159.105. 14385825

[20] Mosaheb MM, Dobrikova EY, Brown MC, Yang Y, Cable J, Okada H, Nair SK, Bigner DD, Ashley DM, Gromeier M. 2020. Genetically stable poliovirus vectors activate dendritic cells and prime antitumor CD8 T cell immunity. Nat Commun 11:524. doi:10.1038/s41467-019-13939-z. 31988324 PMC6985231

[21] Dufresne AT, Gromeier M. 2004. A nonpolio enterovirus with respiratory tropism causes poliomyelitis in intercellular adhesion molecule 1 transgenic mice. Proc Natl Acad Sci USA 101:13636–13641. doi:10.1073/pnas.0403998101. 15353596 PMC518806

[22] Andreev DE, Hirnet J, Terenin IM, Dmitriev SE, Niepmann M, Shatsky IN. 2012. Glycyl-tRNA synthetase specifically binds to the poliovirus IRES to activate translation initiation. Nucleic Acids Res 40:5602–5614. doi:10.1093/nar/gks182. 22373920 PMC3384309

[23] Blyn LB, Swiderek KM, Richards O, Stahl DC, Semler BL, Ehrenfeld E. 1996. Poly(rC) binding protein 2 binds to stem-loop IV of the poliovirus RNA 5′ noncoding region: identification by automated liquid chromatography-tandem mass spectrometry. Proc Natl Acad Sci USA 93:11115–11120. doi:10.1073/pnas.93.20.11115. 8855318 PMC38293

[24] Merrill MK, Gromeier M. 2006. The double-stranded RNA binding protein 76:NF45 heterodimer inhibits translation initiation at the rhinovirus type 2 internal ribosome entry site. J Virol 80:6936–6942. doi:10.1128/JVI.00243-06. 16809299 PMC1489066

[25] Pilipenko EV, Pestova TV, Kolupaeva VG, Khitrina EV, Poperechnaya AN, Agol VI, Hellen CU. 2000. A cell cycle-dependent protein serves as a template-specific translation initiation factor. Genes Dev 14:2028–2045. doi:10.1101/gad.14.16.2028. 10950867 PMC316860

[26] Merrill MK, Dobrikova EY, Gromeier M. 2006. Cell-type-specific repression of internal ribosome entry site activity by double-stranded RNA-binding protein 76. J Virol 80:3147–3156. doi:10.1128/JVI.80.7.3147-3156.2006. 16537583 PMC1440377

[27] Lin JY, Brewer G, Li ML. 2015. HuR and Ago2 bind the internal ribosome entry site of enterovirus 71 and promote virus translation and replication. PLoS One 10:e0140291. doi:10.1371/journal.pone.0140291. 26451954 PMC4599798

[28] Parsyan A, Svitkin Y, Shahbazian D, Gkogkas C, Lasko P, Merrick WC, Sonenberg N. 2011. mRNA helicases: the tacticians of translational control. Nat Rev Mol Cell Biol 12:235–245. doi:10.1038/nrm3083. 21427765

[29] Satoh T, Kato H, Kumagai Y, Yoneyama M, Sato S, Matsushita K, Tsujimura T, Fujita T, Akira S, Takeuchi O. 2010. LGP2 is a positive regulator of RIG-I- and MDA5-mediated antiviral responses. Proc Natl Acad Sci USA 107:1512–1517. doi:10.1073/pnas.0912986107. 20080593 PMC2824407

[30] Pestal K, Funk CC, Snyder JM, Price ND, Treuting PM, Stetson DB. 2015. Isoforms of RNA-editing enzyme ADAR1 independently control nucleic acid sensor MDA5-driven autoimmunity and multi-organ development. Immunity 43:933–944. doi:10.1016/j.immuni.2015.11.001. 26588779 PMC4654992

[31] Gromeier M, Bossert B, Arita M, Nomoto A, Wimmer E. 1999. Dual stem loops within the poliovirus internal ribosomal entry site control neurovirulence. J Virol 73:958–964. doi:10.1128/JVI.73.2.958-964.1999. 9882296 PMC103915

[32] Bou-Nader C, Gordon JM, Henderson FE, Zhang J. 2019. The search for a PKR code-differential regulation of protein kinase R activity by diverse RNA and protein regulators. RNA 25:539–556. doi:10.1261/rna.070169.118. 30770398 PMC6467004

[33] Lemaire PA, Anderson E, Lary J, Cole JL. 2008. Mechanism of PKR Activation by dsRNA. J Mol Biol 381:351–360. doi:10.1016/j.jmb.2008.05.056. 18599071 PMC2570377

[34] Romano PR, Garcia-Barrio MT, Zhang X, Wang Q, Taylor DR, Zhang F, Herring C, Mathews MB, Qin J, Hinnebusch AG. 1998. Autophosphorylation in the activation loop is required for full kinase activity in vivo of human and yeast eukaryotic initiation factor 2alpha kinases PKR and GCN2. Mol Cell Biol 18:2282–2297. doi:10.1128/MCB.18.4.2282. 9528799 PMC121479

[35] Patel RC, Stanton P, Sen GC. 1994. Role of the amino-terminal residues of the interferon-induced protein kinase in its activation by double-stranded RNA and heparin. J Biol Chem 269:18593–18598. doi:10.1016/S0021-9258(17)32351-7. 7518438

[36] Dobrikov MI, Dobrikova EY, Gromeier M. 2013. Dynamic regulation of the translation initiation helicase complex by mitogenic signal transduction to eukaryotic translation initiation factor 4G. Mol Cell Biol 33:937–946. doi:10.1128/MCB.01441-12. 23263986 PMC3623071

[37] Marintchev A, Wagner G. 2005. eIF4G and CBP80 share a common origin and similar domain organization: implications for the structure and function of eIF4G. Biochemistry 44:12265–12272. doi:10.1021/bi051271v. 16156639

[38] Kaiser C, Dobrikova EY, Bradrick SS, Shveygert M, Herbert JT, Gromeier M. 2008. Activation of cap-independent translation by variant eukaryotic initiation factor 4G in vivo. RNA 14:2170–2182. doi:10.1261/rna.1171808. 18755839 PMC2553731

[39] Ohlmann T, Rau M, Pain VM, Morley SJ. 1996. The C-terminal domain of eukaryotic protein synthesis initiation factor (eIF) 4G is sufficient to support cap-independent translation in the absence of eIF4E. EMBO J 15:1371–1382. doi:10.1002/j.1460-2075.1996.tb00479.x. 8635470 PMC450042

[40] Wong AH, Tam NW, Yang YL, Cuddihy AR, Li S, Kirchhoff S, Hauser H, Decker T, Koromilas AE. 1997. Physical association between STAT1 and the interferon-inducible protein kinase PKR and implications for interferon and double-stranded RNA signaling pathways. EMBO J 16:1291–1304. doi:10.1093/emboj/16.6.1291. 9135145 PMC1169727

[41] Goh KC, deVeer MJ, Williams BR. 2000. The protein kinase PKR is required for p38 MAPK activation and the innate immune response to bacterial endotoxin. EMBO J 19:4292–4297. doi:10.1093/emboj/19.16.4292. 10944112 PMC302024

[42] Taghavi N, Samuel CE. 2012. Protein kinase PKR catalytic activity is required for the PKR-dependent activation of mitogen-activated protein kinases and amplification of interferon beta induction following virus infection. Virology 427:208–216. doi:10.1016/j.virol.2012.01.029. 22381929 PMC3313008

[43] Bonnet MC, Weil R, Dam E, Hovanessian AG, Meurs EF. 2000. PKR stimulates NF-kappaB irrespective of its kinase function by interacting with the IkappaB kinase complex. Mol Cell Biol 20:4532–4542. doi:10.1128/MCB.20.13.4532-4542.2000. 10848580 PMC85837

[44] Gil J, Alcami J, Esteban M. 2000. Activation of NF-kappa B by the dsRNA-dependent protein kinase, PKR involves the I kappa B kinase complex. Oncogene 19:1369–1378. doi:10.1038/sj.onc.1203448. 10723127

[45] Zamanian-Daryoush M, Mogensen TH, DiDonato JA, Williams BR. 2000. NF-kappaB activation by double-stranded-RNA-activated protein kinase (PKR) is mediated through NF-kappaB-inducing kinase and IkappaB kinase. Mol Cell Biol 20:1278–1290. doi:10.1128/MCB.20.4.1278-1290.2000. 10648614 PMC85265

[46] Kok KH, Lui PY, Ng MH, Siu KL, Au SW, Jin DY. 2011. The double-stranded RNA-binding protein PACT functions as a cellular activator of RIG-I to facilitate innate antiviral response. Cell Host Microbe 9:299–309. doi:10.1016/j.chom.2011.03.007. 21501829

[47] Sanchez David RY, Combredet C, Najburg V, Millot GA, Beauclair G, Schwikowski B, Leger T, Camadro JM, Jacob Y, Bellalou J, Jouvenet N, Tangy F, Komarova AV. 2019. LGP2 binds to PACT to regulate RIG-I- and MDA5-mediated antiviral responses. Sci Signal 12:eaar3993. doi:10.1126/scisignal.aar3993. 31575732

[48] Chukwurah E, Farabaugh KT, Guan BJ, Ramakrishnan P, Hatzoglou M. 2021. A tale of two proteins: PACT and PKR and their roles in inflammation. FEBS J 288:6365–6391. doi:10.1111/febs.15691. 33387379 PMC9248962

[49] Kastan JP, Dobrikova EY, Bryant JD, Gromeier M. 2020. CReP mediates selective translation initiation at the endoplasmic reticulum. Sci Adv 6:eaba0745. doi:10.1126/sciadv.aba0745. 32537501 PMC7269655

[50] Kato H, Takeuchi O, Sato S, Yoneyama M, Yamamoto M, Matsui K, Uematsu S, Jung A, Kawai T, Ishii KJ, Yamaguchi O, Otsu K, Tsujimura T, Koh CS, Reis e Sousa C, Matsuura Y, Fujita T, Akira S. 2006. Differential roles of MDA5 and RIG-I helicases in the recognition of RNA viruses. Nature 441:101–105. doi:10.1038/nature04734. 16625202

[51] Brown MC, Bryant JD, Dobrikova EY, Shveygert M, Bradrick SS, Chandramohan V, Bigner DD, Gromeier M. 2014. Induction of viral, 7-methyl-guanosine cap-independent translation and oncolysis by mitogen-activated protein kinase-interacting kinase-mediated effects on the serine/arginine-rich protein kinase. J Virol 88:13135–13148. doi:10.1128/JVI.01883-14. 25187541 PMC4249076

[52] Brown MC, Dobrikov MI, Gromeier M. 2014. Mitogen-activated protein kinase-interacting kinase regulates mTOR/AKT signaling and controls the serine/arginine-rich protein kinase-responsive type 1 internal ribosome entry site-mediated translation and viral oncolysis. J Virol 88:13149–13160. doi:10.1128/JVI.01884-14. 25187540 PMC4249080

[53] Goetz C, Everson RG, Zhang LC, Gromeier M. 2010. MAPK signal-integrating kinase controls cap-independent translation and cell type-specific cytotoxicity of an oncolytic poliovirus. Mol Ther 18:1937–1946. doi:10.1038/mt.2010.145. 20648000 PMC2990508

[54] Walton RW, Brown MC, Sacco MT, Gromeier M. 2018. Engineered oncolytic poliovirus PVSRIPO subverts MDA5-dependent innate immune responses in cancer cells. J Virol 92:e00879-18. doi:10.1128/JVI.00879-18. 29997212 PMC6146809

[55] Ahmad S, Mu X, Yang F, Greenwald E, Park JW, Jacob E, Zhang CZ, Hur S. 2018. Breaching self-tolerance to Alu duplex RNA underlies MDA5-mediated inflammation. Cell 172:797–810.e13. doi:10.1016/j.cell.2017.12.016. 29395326 PMC5807104

[56] Chung H, Calis JJA, Wu X, Sun T, Yu Y, Sarbanes SL, Dao Thi VL, Shilvock AR, Hoffmann HH, Rosenberg BR, Rice CM. 2018. Human ADAR1 prevents endogenous RNA from triggering translational shutdown. Cell 172:811–824.e14. doi:10.1016/j.cell.2017.12.038. 29395325 PMC5831367

[57] Pfaller CK, Li Z, George CX, Samuel CE. 2011. Protein kinase PKR and RNA adenosine deaminase ADAR1: new roles for old players as modulators of the interferon response. Curr Opin Immunol 23:573–582. doi:10.1016/j.coi.2011.08.009. 21924887 PMC3190076

[58] Dobrikov MI, Shveygert M, Brown MC, Gromeier M. 2014. Mitotic phosphorylation of eukaryotic initiation factor 4G1 (eIF4G1) at Ser1232 by Cdk1:cyclin B inhibits eIF4A helicase complex binding with RNA. Mol Cell Biol 34:439–451. doi:10.1128/MCB.01046-13. 24248602 PMC3911517

[59] Dobrikov MI, Dobrikova EY, Gromeier M. 2018. Ribosomal RACK1:PKCbetaII modulates intramolecular interactions between unstructured regions of eIF4G that control eIF4E and eIF3 binding. Mol Cell Biol 38:e00306-18. doi:10.1128/MCB.00306-18. 30012864 PMC6146829

[60] Dobrikov MI, Dobrikova EY, Gromeier M. 2018. Ribosomal RACK1:PKCbetaII phosphorylates eIF4G1(S1093) to modulate cap-dependent and -independent translation initiation. Mol Cell Biol 38:e00304-18. doi:10.1128/MCB.00304-18. 30012863 PMC6146833

