# Supplementary figures and images for: PKR Binds Enterovirus IRESs, Displaces Host Translation Factors, and Impairs Viral Translation to Enable Innate Antiviral Signaling
Source: mBio. 2022 Jun 2;13(3):e00854-22. doi: 10.1128/mbio.00854-22 (PMC9239082; doi:10.1128/mbio.00854-22)

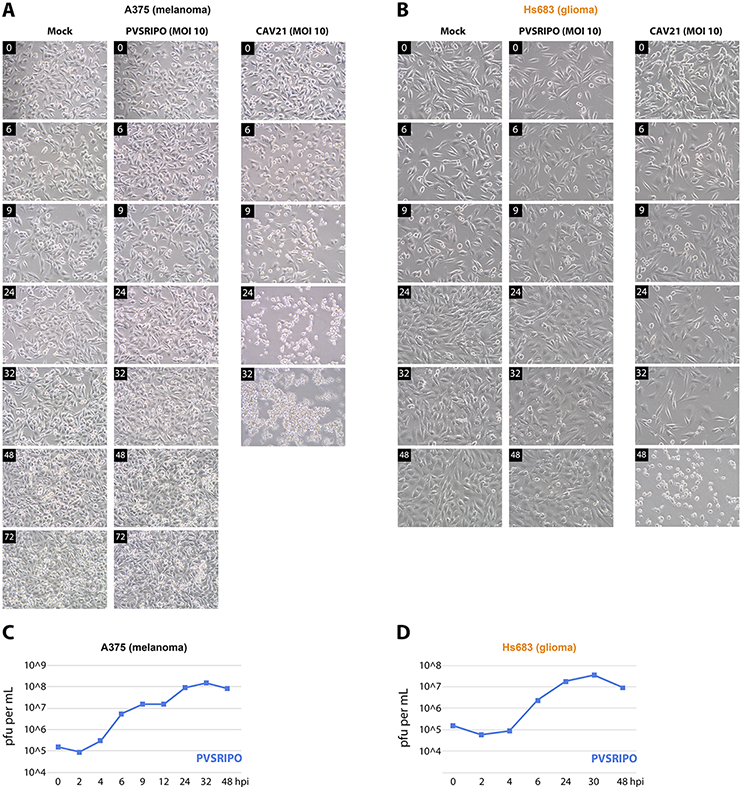

Supplement: FIG S1 [file mbio.00854-22-s0004.tif]

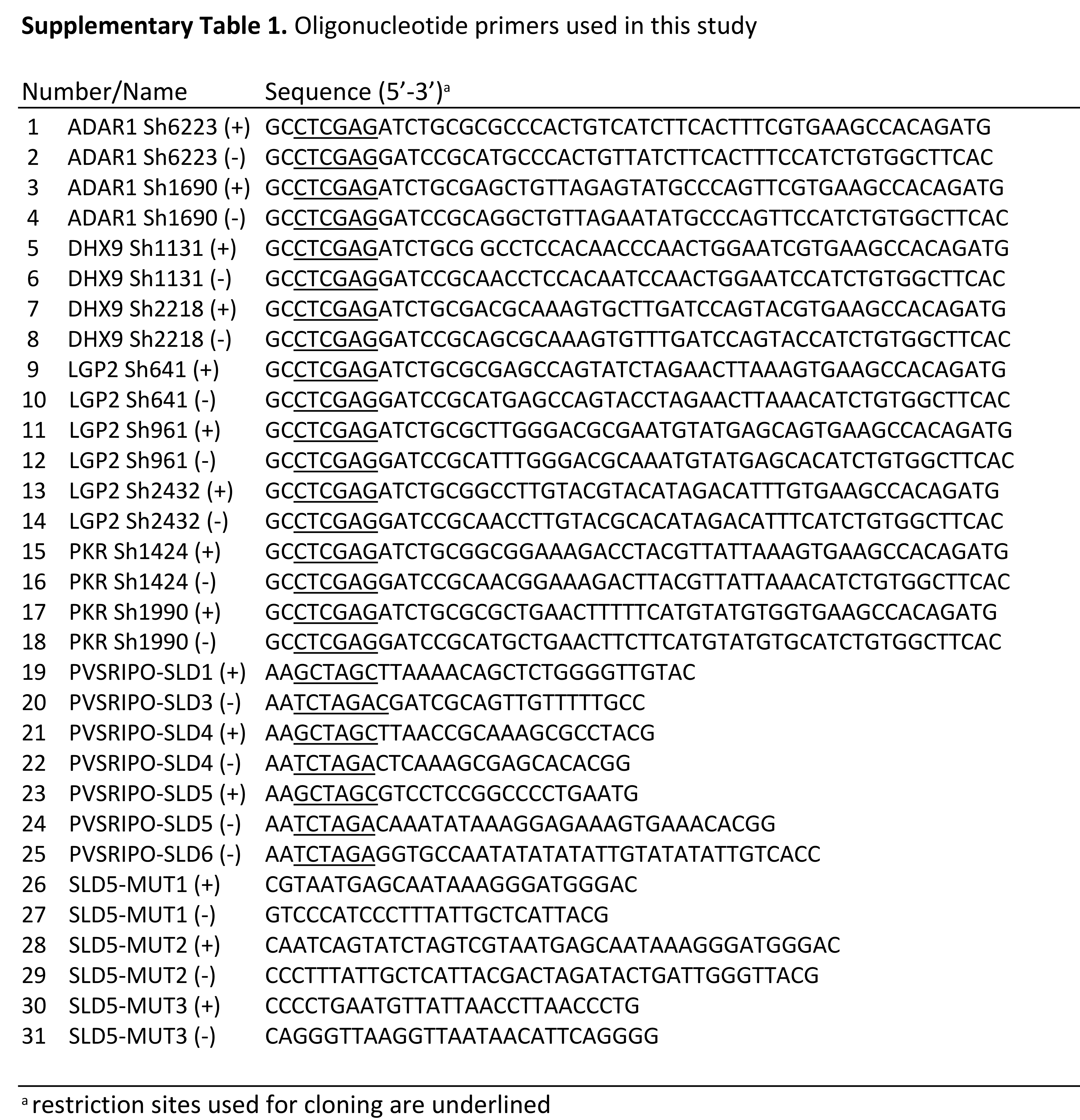

Supplement: TABLE S1 [file mbio.00854-22-s0001.tif]

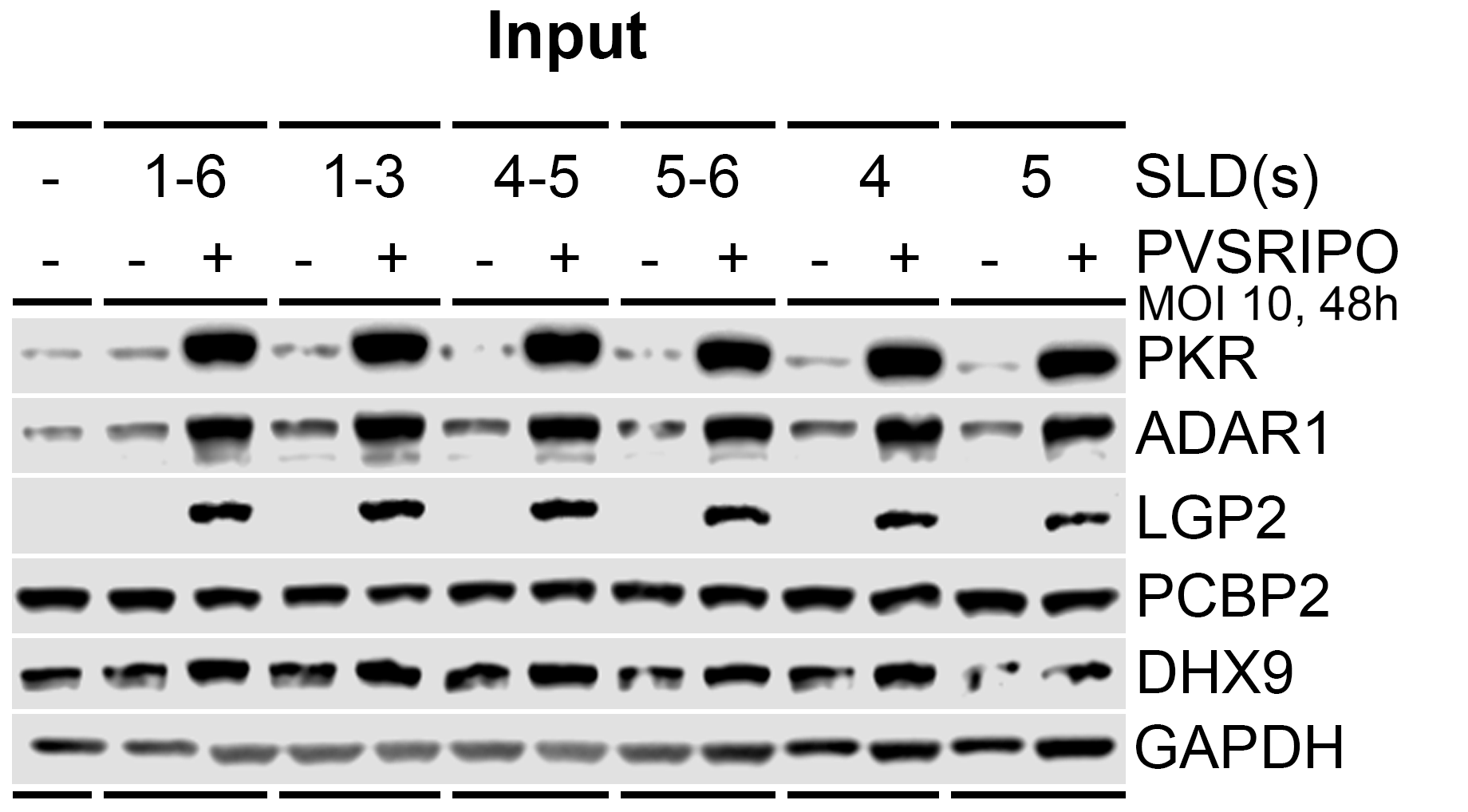

Supplement: FIG S2 [file mbio.00854-22-s0005.tif]

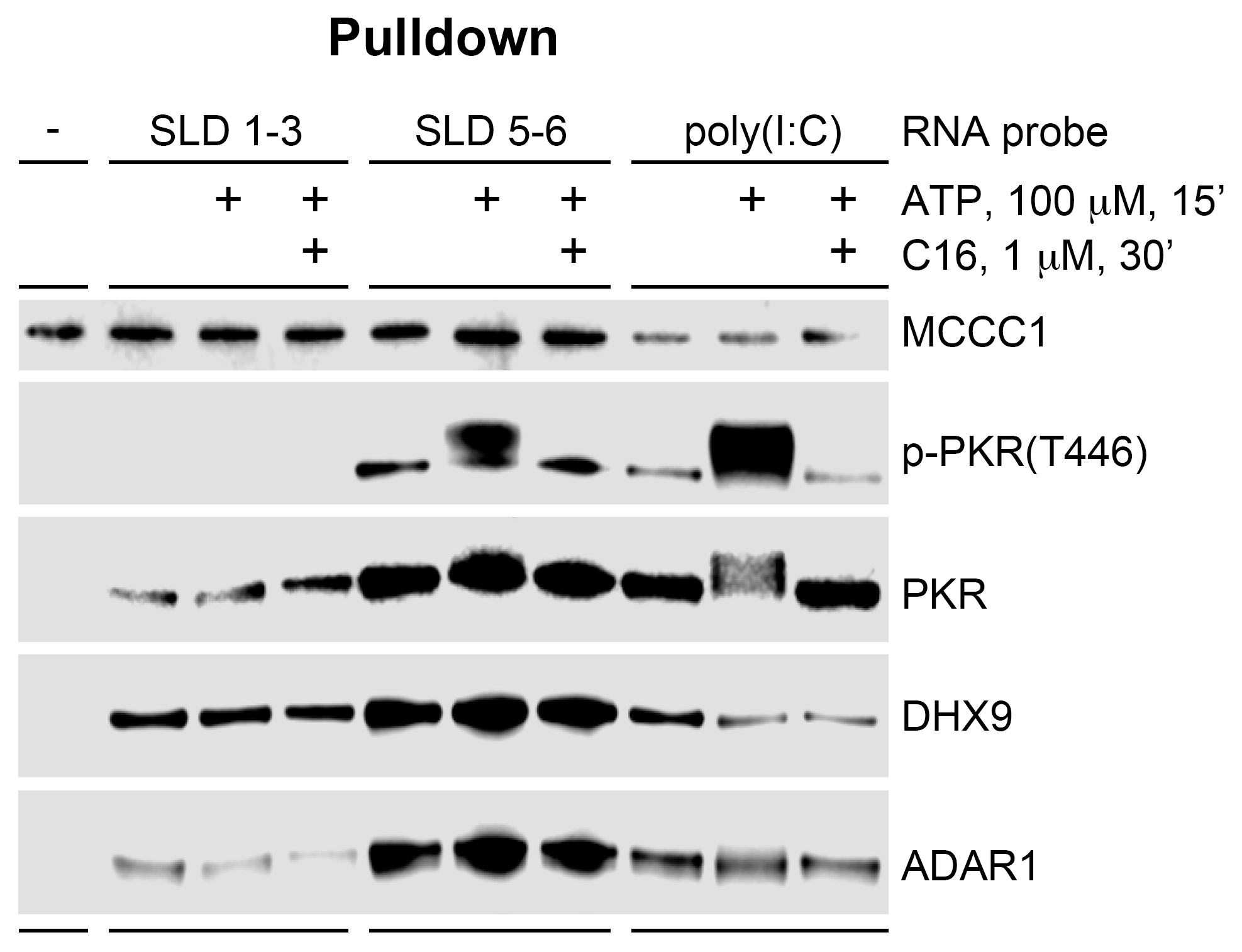

Supplement: FIG S3 [file mbio.00854-22-s0006.tif]

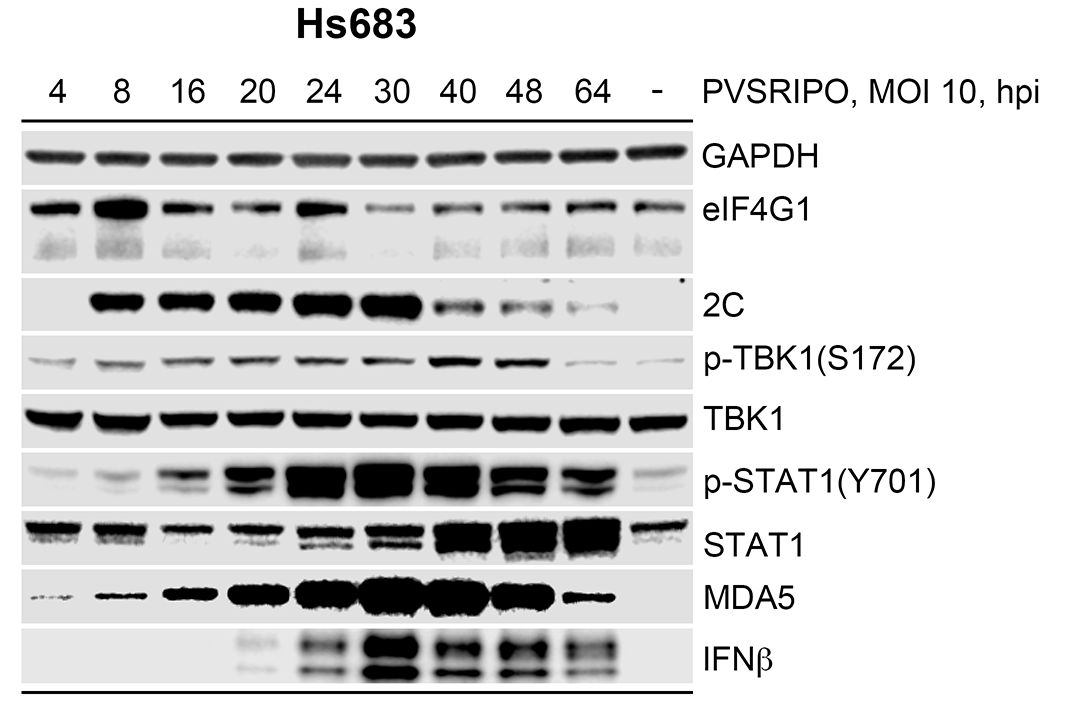

Supplement: FIG S4 [file mbio.00854-22-s0007.tif]

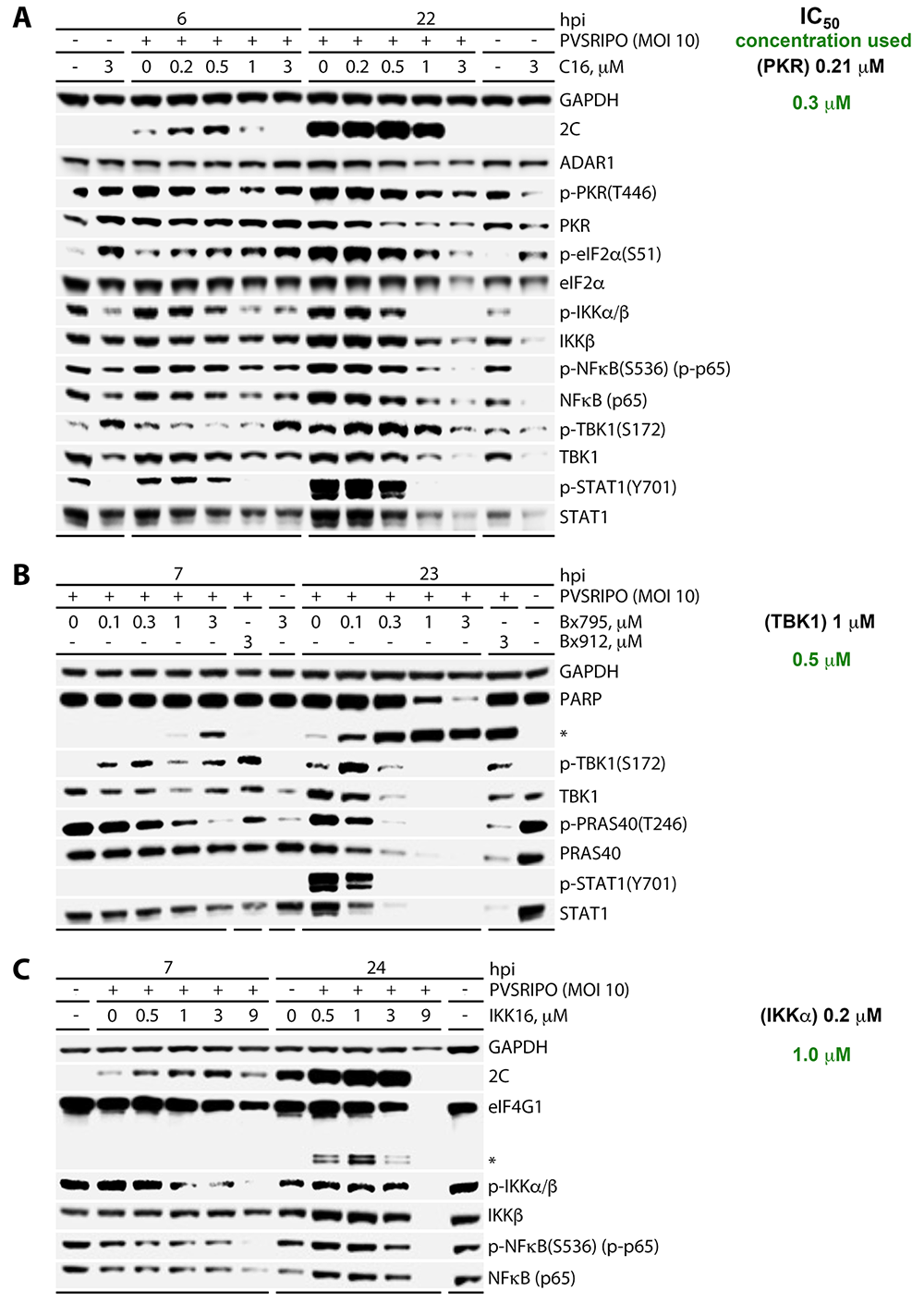

Supplement: FIG S5 [file mbio.00854-22-s0008.tif]

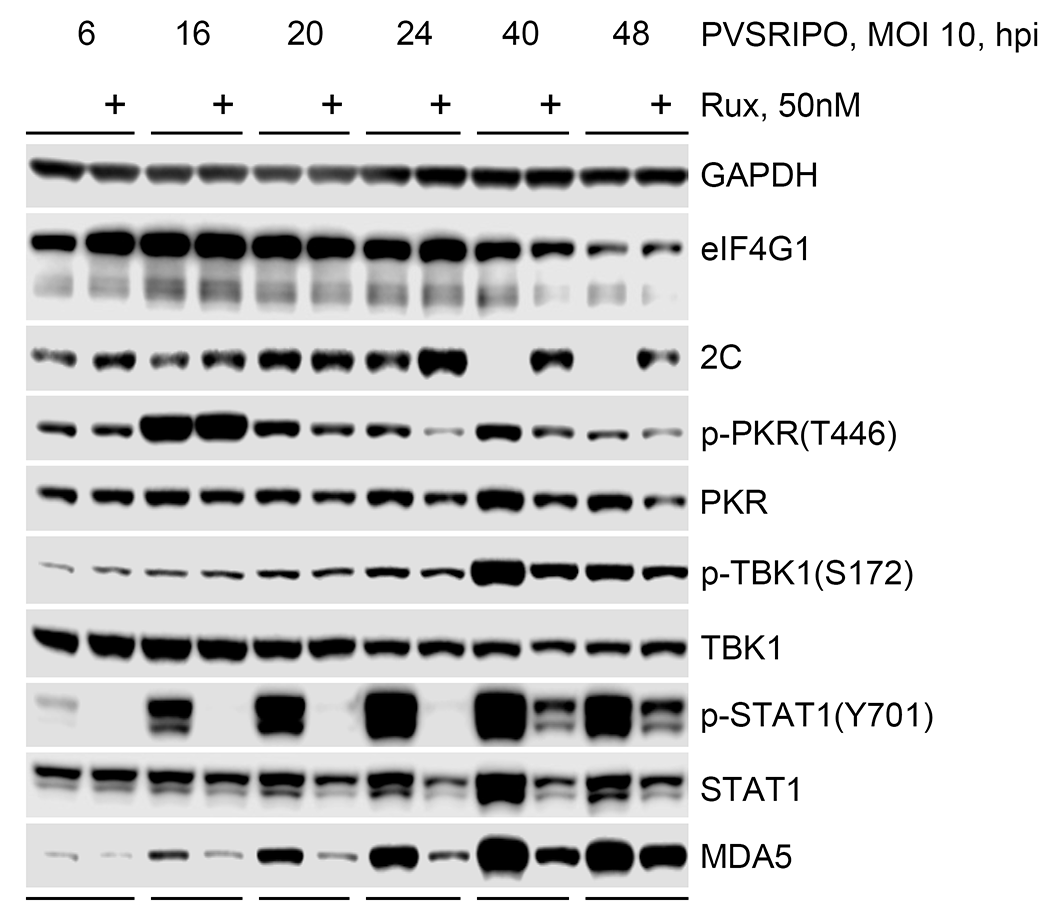

Supplement: FIG S7 [file mbio.00854-22-s0010.tif]

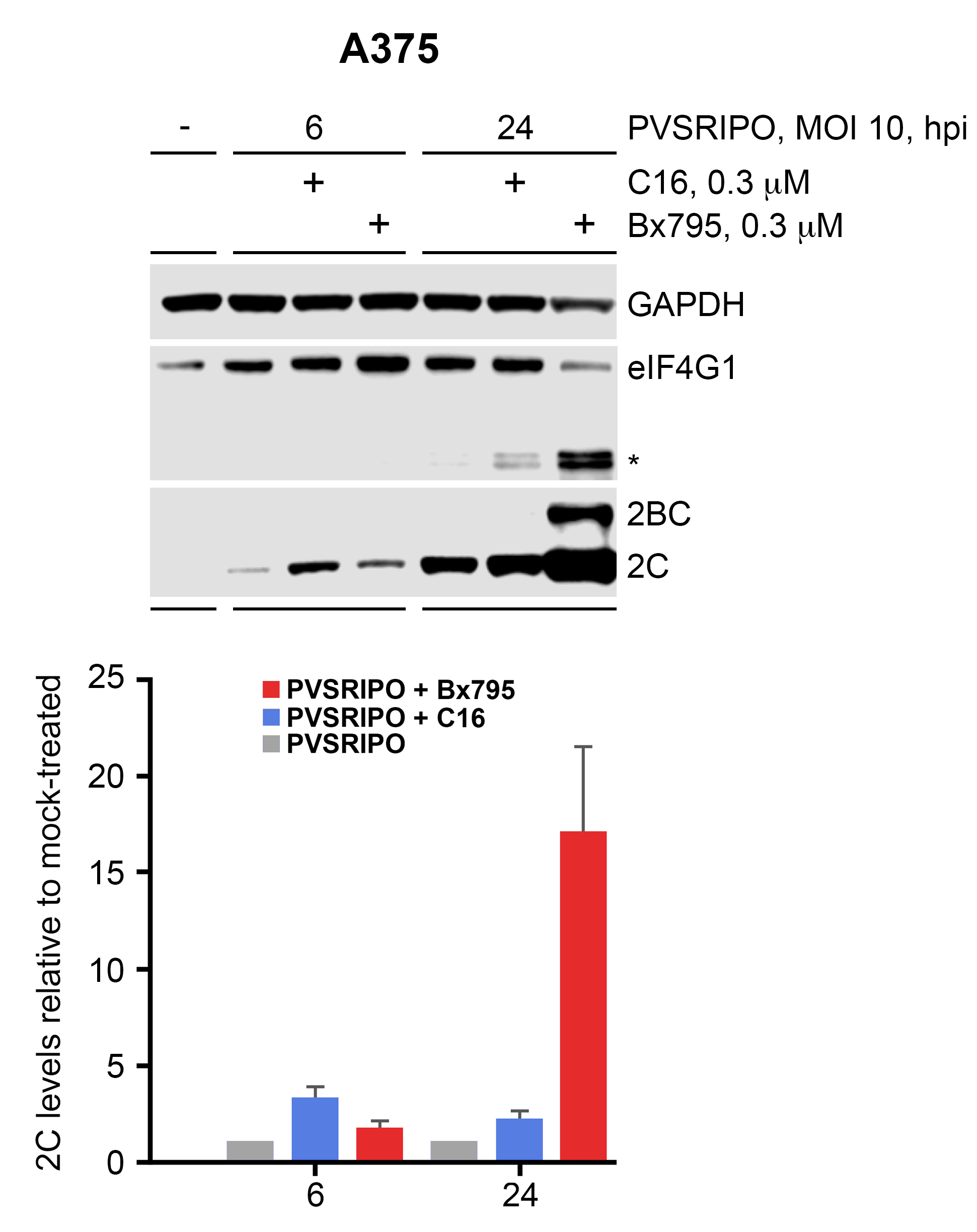

Supplement: FIG S6 [file mbio.00854-22-s0009.tif]
